# Interplay between the RNA Chaperone Hfq, Small RNAs and Transcriptional Regulator OmpR Modulates Iron Homeostasis in the Enteropathogen *Yersinia enterocolitica*

**DOI:** 10.3390/ijms241311157

**Published:** 2023-07-06

**Authors:** Karolina Jaworska, Julia Konarska, Patrycja Gomza, Paula Rożen, Marta Nieckarz, Agata Krawczyk-Balska, Katarzyna Brzostek, Adrianna Raczkowska

**Affiliations:** Department of Molecular Microbiology, Institute of Microbiology, Faculty of Biology, University of Warsaw, Miecznikowa 1, 02-096 Warsaw, Poland

**Keywords:** *Yersinia enterocolitica*, RNA chaperone Hfq, Fur repressor, sRNA RyhB1, sRNA OmrA, regulator OmpR, iron homeostasis, *fur*, *fecA*, *fepA*

## Abstract

Iron is both essential for and potentially toxic to bacteria, so the precise maintenance of iron homeostasis is necessary for their survival. Our previous study indicated that in the human enteropathogen *Yersinia enterocolitica*, the regulator OmpR directly controls the transcription of the *fur*, *fecA* and *fepA* genes, encoding the ferric uptake repressor and two transporters of ferric siderophores, respectively. This study was undertaken to determine the significance of the RNA chaperone Hfq and the small RNAs OmrA and RyhB1 in the post-transcriptional control of the expression of these OmpR targets. We show that Hfq silences *fur*, *fecA* and *fepA* expression post-transcriptionally and negatively affects the production of FLAG-tagged Fur, FecA and FepA proteins. In addition, we found that the *fur* gene is under the negative control of the sRNA RyhB1, while *fecA* and *fepA* are negatively regulated by the sRNA OmrA. Finally, our data revealed that the role of OmrA results from a complex interplay of transcriptional and post-transcriptional effects in the feedback circuit between the regulator OmpR and the sRNA OmrA. Thus, the expression of *fur*, *fecA* and *fepA* is subject to complex transcriptional and post-transcriptional regulation in order to maintain iron homeostasis in *Y. enterocolitica*.

## 1. Introduction

The concentration of iron in the environment is critical for the control of bacterial metabolism and physiology [1,2]. Iron limitation can abolish bacterial growth, whereas a high intracellular iron concentration may be deleterious due to the generation of harmful reactive oxygen species [3]. Thus, the ability to maintain iron homeostasis is an important element in the functioning of bacteria, and the expression of genes involved in the transport and storage of iron has to be tightly controlled [4,5].

Multiple iron acquisition systems operate in bacteria for the uptake of iron from the environment [6,7,8]. The best characterized of these are based on the use of siderophores: low-molecular-weight chelators with a high affinity for ferric iron (Fe^3+^) ions. The characteristic feature of siderophores is their ability to capture ferric iron from the environment and transport it within the resulting complex into the bacterial cytosol via specific outer membrane (OM) receptors/transporters in a TonB-dependent manner [9]. Essentially, all genes encoding siderophore-based iron transport systems in enterobacteria are controlled by the Fur protein (ferric uptake regulator), an Fe^2+^-responsive transcription factor, the major regulator of iron homeostasis in these bacteria [4]. The expression of *fur* in *Escherichia coli* is controlled by several transcriptional regulators [10]. Another important layer of *fur* regulation in *E. coli* is post-transcriptional downregulation involving the small non-coding RNA (sRNA) RyhB [11].

*Yersinia enterocolitica* is the causative agent of yersiniosis, an enteric infection with symptoms including gastroenteritis, watery diarrhea and mesenteric lymphadenitis [12,13]. Yersiniosis is the third most commonly reported gastrointestinal infection in the EU/EEA after campylobacteriosis and salmonellosis [14]. *Y. enterocolitica* uses a variety of ways to obtain iron from the surrounding environment, including siderophore-mediated uptake and systems for acquiring iron from abundant heme or hemoproteins [15,16,17]. Pathogenic *Y. enterocolitica* strains can be divided into highly pathogenic members of biotype 1B, producing the siderophore yersiniabactin encoded on the chromosomal pathogenicity island HPI [18], and less pathogenic strains of biotypes 2–5 that are unable to produce this siderophore but can import foreign ferric siderophores via TonB-dependent OM transporters/receptors such as FecA (ferric citrate), FepA (ferric enterobactin, also known as ferrienterochelin), FoxA (ferrioxamine) and FcuA (ferrichrome) [19,20]. Almost all of the iron-scavenging mechanisms in *Yersiniae* are controlled by Fur [19,20,21,22].

Bacterial sRNAs act as key regulators of many cellular functions by modulating gene expression in response to various environmental signals [23,24,25,26,27,28,29]. This regulation is thought to allow bacteria to rapidly adapt to changes in the environment. The majority of bacterial sRNAs are *trans*-encoded, 50–150 nt in length, and form short imperfect base-pairing interactions with complementary sequences in their mRNA targets. The interaction of an sRNA with the mRNA target can positively or negatively impact gene expression at the level of translation initiation, mRNA stability or transcription termination [23]. Studies on *Yersiniae* have identified numerous sRNAs, including homologs of those found in *E. coli* [30,31,32,33]. A fairly high resolution transcriptome analysis revealed repertoires of 204 and 119 sRNAs expressed from the intergenic regions of *Y. enterocolitica* strains 8081v (bioserotype 1B/O:8) and Y1 (bioserotype 4/O:3), respectively [34]. Although comprehensive expression profiles of the identified sRNAs have been presented, the interaction partners and biological function of many of these *trans*-encoded sRNAs have yet to be identified.

Since the sRNA-binding sites on their mRNA targets are typically short (6–8 contiguous base pairs) and imperfect, many sRNAs in enterobacteria require the RNA chaperone Hfq for their stability and function [35,36,37]. Hfq, first described as a host factor required for the replication of the Qβ phage in *E. coli* [38], is a widely conserved protein that stabilizes sRNAs, facilitates sRNA-mRNA base pairing and modulates the degradation of target RNAs [23,37,39]. Homologs of *E. coli* Hfq have been described in many bacteria, including pathogenic *Yersiniae* [40]. It appears that Hfq, probably in combination with sRNAs, can control the expression of many virulence- and stress-associated genes in pathogenic bacteria [41], including *Y. pestis* [42], *Y. pseudotuberculosis* [43] and *Y. enterocolitica* [44,45].

Interestingly, out of ~100 sRNAs identified in *E. coli*, around 30 have been shown to be Hfq-dependent [23,39,46]. These include RyhB [29], which, in addition to its important role in maintaining iron homeostasis [47,48,49], acts as a regulator of various processes, such as biofilm formation and chemotaxis [50,51], acid resistance [52] and the repression of virulence genes [53]. Moreover, the homologous sRNAs OmrA and OmrB were discovered during two genome-wide searches in *E. coli* [54,55]. Both of these sRNAs participate in controlling the expression of a number of target mRNAs, including those encoding several OM proteins: protease OmpT and receptors for the ferric siderophores FepA, FecA and CirA [24,56]. OmrA and OmrB also repress the synthesis of some transcriptional regulators, such as CsgD for curli formation [57] and FlhDC for flagella biogenesis [58,59]. The mode of action of *E. coli* OmrA/B sRNAs has been studied in detail. The 5′ ends of these sRNAs are involved in the regulation of mRNA targets by base pairing, with the participation of Hfq and RNase E [56]. Interestingly, these sRNAs autoregulate their transcription in *E. coli* by a feedback control mechanism involving the two-component system (TCS) EnvZ/OmpR [24,60]. This system, comprising the EnvZ transmembrane sensor kinase and the transcriptional response regulator OmpR, modulates the expression of numerous genes in response to environmental changes such as altered osmolarity, pH and nutrient availability [61,62,63]. A large body of research has demonstrated that OmpR influences a wide variety of cellular processes in *E. coli* [64,65,66], *Salmonella* and *Shigella* [67,68], as well as pathogenic *Yersinia* species [69,70,71,72,73,74].

We recently reported that the OmpR regulator in *Y. enterocolitica* strain Ye9N (bioserotype 2/O:9) modulates the expression of *fur*, as well as the genes *fecA* and *fepA* (members of the Fur regulon), at the transcriptional level by directly binding to their promoters [22]. The objective of the present study was to unravel an additional post-transcriptional layer of control in the expression of these OmpR targets. Our investigation focused on the significance of the RNA chaperone Hfq in the production of the Fur repressor and the ferric siderophore transporters FecA and FepA. It was also intriguing to check whether the sRNA RyhB1 homolog is involved in the post-transcriptional regulation of *fur*. As only one gene with homology to *E. coli omrA* and *omrB* has been identified in the genomes of *Yersiniae* [30,31], this raised the question of whether this sRNA participates in the post-transcriptional regulation of *fur* or *fecA* and *fepA* in *Y. enterocolitica*. It was also interesting to check whether the regulatory loop between the Omr sRNAs and OmpR observed in *E. coli* exists in *Y. enterocolitica* and, if so, whether it exerts any effect on *fecA* and *fepA* expression.

## 2. Results

### 2.1. The RNA Chaperone Hfq and sRNA RyhB1 Impact Fur Expression in Y. enterocolitica 2/O:9, Strain Ye9N

To examine the role of Hfq in the expression of genes involved in iron uptake and regulation in *Y. enterocolitica* strain Ye9N (bioserotype 2/O:9), an isogenic Δ*hfq* mutant lacking the Hfq-coding sequence was generated (strain Ye9*hfq*) (see Materials and Methods). Hfq typically acts with sRNAs to modulate mRNA stability and/or translation; thus, direct regulation by Hfq is usually dependent upon base pairing between the sRNA and its target mRNA near the ribosome-binding site. Bearing this in mind, the role of Hfq was tested using a plasmid carrying a translational fusion of *fur* with the *gfp* reporter gene. This plasmid, pFX-fur, was introduced into Ye9*hfq* and the wild-type strain Ye9N. In addition, the effect of the complementation of the Δ*hfq* mutation or the overexpression of Hfq in the wild-type strain was tested by introducing a native copy of *hfq* under the control of the arabinose-inducible P*_BAD_* promoter in the plasmid pBAD-Hfq, with the empty vector pBAD24Cm as a control. These constructs are compatible with pFX plasmids, so they can be stably maintained together in the cell. To reveal the role of Hfq in iron-dependent *fur* regulation in *Y. enterocolitica*, the strains were grown in LB medium and under iron-starved conditions, i.e., LB supplemented with 150 µM dipyridyl, an iron chelator (LBD medium). It was previously suggested that iron may be involved in the regulation of *fur* transcription in *E. coli* [75] and *Y. enterocolitica* [22].

**Figure 1:**
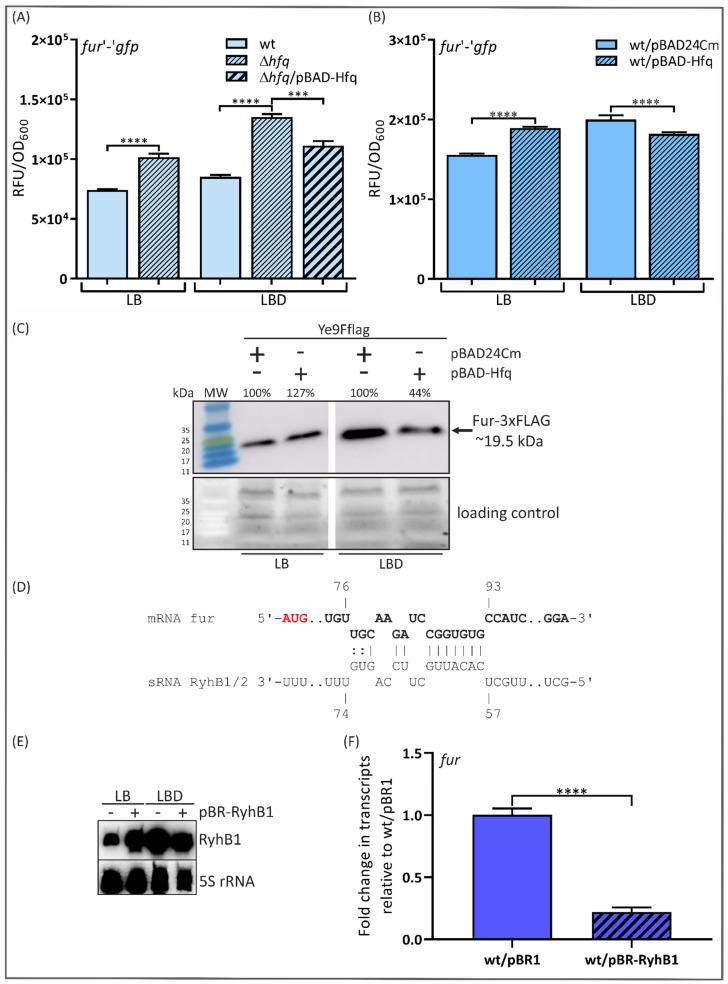
Post-transcriptional regulation of *fur* expression. (**A**,**B**) Influence of Hfq on *fur* expression. Green fluorescence of strain Ye9N and its derivatives carrying a *fur*′-′*gfp* translational fusion (pFX-fur) was measured in cultures grown to the stationary phase in LB or LBD. The presented data are the mean relative fluorescence values normalized to the OD_600_ of the culture (±standard deviation) from at least three independent experiments, each performed with three separate cultures per strain. Significance was calculated by one-way ANOVA (**** *p* < 0.0001, *** *p* < 0.001). (**C**) Influence of Hfq on the abundance of Fur-3×FLAG protein. Levels of Fur-3×FLAG were analyzed in Ye9Fflag cells carrying either the empty vector pBAD24Cm or the plasmid pBAD-Hfq, grown to the stationary phase in LB or LBD. Western blotting of cell lysates was performed with a monoclonal mouse anti-FLAG epitope antibody. The upper panel shows the immunoblot, and the lower panel shows part of the Bio-Rad Stain-Free gel as a loading control. The percentage values indicate the immunostained protein band intensities relative to Ye9Fflag/pBAD24Cm grown in LB or LBD. MW—color prestained protein marker. (**D**) Predicted base pairing between sRNAs RyhB1/RyhB2 and the *fur* transcript. The mRNA sequences are in bold, with the translation start codons marked in red. Nucleotides are numbered from the first base of the start codon. (**E**) Northern blot showing the level of *ryhB1* mRNA in strains Ye9N (wt) and Ye9N/pBR-RyhB1 grown in LB or LBD. As a loading control, the level of 5S rRNA was examined. (**F**) The level of *fur* transcripts assessed by RT-qPCR in Ye9N (wt) and Ye9N/pBR-RyhB1 grown to early stationary phase in LBD. Relative *fur* transcript levels, normalized to the amount of 16S rRNA, are shown, taking the mRNA level in Ye9N/pBR1 as 1. The presented data are the mean values (±standard deviation) obtained from at least three independent experiments. Significance was calculated using Student’s unpaired *t*-test (**** *p* < 0.0001).

As shown in Figure 1A, the green fluorescence of Ye9*hfq*/pFX-fur increased by 27 and 37% relative to the parental strain Ye9N/pFX-fur in LB and LBD media, i.e., regardless of the iron concentration. The plasmid-borne copy of the *hfq* gene (pBAD-Hfq) introduced into Ye9*hfq*/pFX-fur grown in LBD only slightly decreased *fur* expression (~15%). Interesting data were obtained using the wild-type strain Ye9N/pFX-fur carrying the plasmid pBAD-Hfq. Hfq overexpression caused a slight increase in green fluorescence in the presence of abundant iron (LB medium) and decreased fluorescence under iron-starved conditions (LBD) (Figure 1B). These significant and opposite alterations in *fur* expression observed under iron-replete and iron-limiting conditions in the presence of increased Hfq are likely to reflect changes resulting from other putative regulatory factors/mechanisms whose activity is triggered by iron and depends on the level of Hfq. These results indicated that the link between Fur and Hfq might be more complex.

To further examine the role of Hfq in the iron-dependent regulation of *fur*, the abundance of the Fur protein was assessed using strain Ye9Fflag, which carries a chromosomal copy of a gene encoding an in-frame translational fusion of Fur with a 3×FLAG epitope peptide at the C-terminal end [22]. Plasmids pBAD24Cm and pBAD-Hfq were introduced into Ye9Fflag, and the obtained strains were grown in LB medium or under iron-limiting conditions (LBD). The Fur-3×FLAG protein was detected in whole-cell lysates by Western blotting using an anti-FLAG antibody. As shown in Figure 1C, the epitope-tagged Fur protein displayed strong and specific reactivity with this antibody. Interestingly, the amount of Fur-3×FLAG in the strain carrying pBAD-Hfq was increased (~27%) in LB medium and decreased (~56%) under iron-limiting conditions (LBD medium) compared with the strain carrying pBAD24Cm (Figure 1C). This opposite effect of Hfq on *fur* expression in iron-replete and iron-limiting media is consistent with the results obtained using the *fur*′-′*gfp* translational fusion under the same growth conditions. In addition, the amount of Fur-3×FLAG increased (~130%) in strain Ye9Fflag/pBAD24Cm in LBD compared with LB medium, confirming the importance of iron in regulating *fur* expression [22]. In summary, Fur abundance in *Y. enterocolitica* is modulated by the level of Hfq, and iron plays a role in this regulation.

Since the main function of Hfq is to act as a cofactor of sRNAs, Hfq/sRNA circuits might directly affect *fur* gene expression, and the interaction of Hfq/RyhB homologs may be responsible for the iron-dependent negative regulation of *fur* [11]. However, indirect regulation is also possible because Hfq could modulate the level of potential activator or repressor proteins involved in *fur* regulation. To study the effect of Hfq overexpression in a time-dependent manner, the Fur-3×FLAG level was monitored following *hfq* induction (30, 60, 90, 120 min after induction). However, there were no changes in the Fur protein level, which indicated both the direct and indirect Hfq-dependent regulation of *fur*.

It was previously determined that the sRNA RyhB, whose expression is negatively regulated by iron (Fe^2+^-Fur), may play a role in the control of *fur* expression in *E. coli* [11]. Interestingly, *Yersinia* species encode two RyhB homologs, named RyhB1 and RyhB2 [76,77]. The nucleotide sequences of the *ryhB1* (108 nt) and *ryhB2* (109 nt) genes in *Y. enterocolitica* are 64% and 63% identical to those of the *E. coli* homologs, respectively. By applying the IntaRNA program (version 5.0.7) to predict interactions between RNA molecules, we identified potential binding sites for both sRNAs within the *fur* mRNA (Figure 1D) and an additional target for base pairing with RyhB2 at the start of the coding sequence. To test whether either of these sRNAs is involved in the regulation of *fur* expression in *Y. enterocolitica*, the plasmid pBR-RyhB1 was created by cloning the Ye9N *ryhB1* gene into the expression vector pBR-plac, named pBR1 (a kind gift from Prof. Susan Gottesman) [24]. Initially, we examined the abundance of *ryhB1* in strains Ye9N and Ye9N/pBR-RyhB1 grown in LB or LBD by Northern blotting. The applied probe was designed to be specific for *ryhB1* so will not bind to *ryhB2*. As shown in Figure 1E, the level of the *ryhB1* transcript was increased in the wild-type strain grown in LBD compared to LB, suggesting the influence of iron on *ryhB1* expression. Interestingly, the *ryhB1* transcript was also present in strain Ye9N grown in LB, which may explain the regulatory effect of Hfq on *fur* expression under iron-replete conditions (Figure 1A). The *ryhB1* mRNA was strikingly more abundant in Ye9N carrying the plasmid pBR-RyhB1. Moreover, the level of the *ryhB1* transcript in this strain was slightly reduced under iron-limiting conditions. This decrease may result from specific degradation mechanisms that regulate the levels of sRNAs [78].

To determine the role of RyhB1 in regulating *fur* expression, RT-qPCR was performed (Figure 1F). The *fur* transcript level was significantly decreased by the presence of plasmid-borne RyhB1, suggesting a negative role for RyhB1 in the post-transcriptional regulation of *fur* in *Y. enterocolitica.* RyhB2 might play a similar role in *fur* regulation, but this has yet to be confirmed.

### 2.2. The Expression of fecA and fepA Is Silenced by Hfq

**Figure 2:**
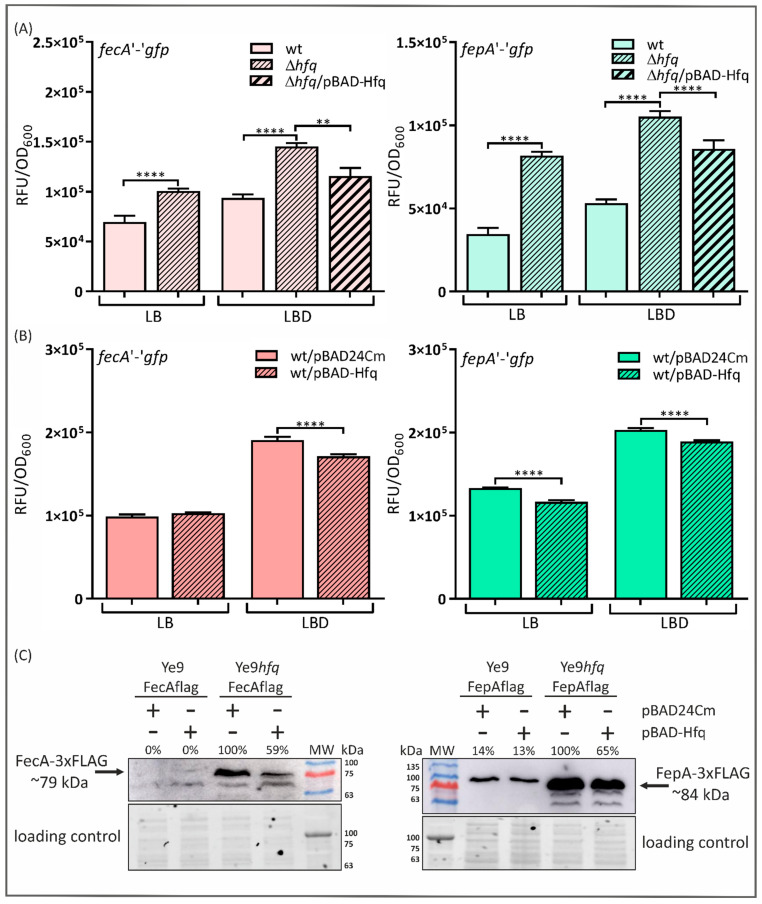
Influence of Hfq on *fecA* and *fepA* expression. (**A**,**B**) Green fluorescence of strain Ye9N and its derivatives carrying a *fecA*’-’*gfp* translational fusion (pFX-*fecA*) or *fepA*’-’*gfp* translational fusion (pFX-*fepA*) measured in cultures grown to stationary phase in LB or LBD medium. The presented data are the mean relative fluorescence values normalized to the OD_600_ of the culture (±standard deviation) from at least three independent experiments, each performed with three separate cultures per strain. Significance was calculated by one-way ANOVA (**** *p* < 0.0001, ** *p* < 0.01). (**C**) Influence of Hfq on FecA and FepA abundance in *Y. enterocolitica* strains. Levels of FecA-3×FLAG (**left panel**) and FepA-3×FLAG (**right panel**) in strains Ye9N and Ye9*hfq* carrying plasmid pBAD-Hfq or empty vector pBAD24Cm grown to stationary phase in LBD. The percentage values indicate the immunostained protein band intensities relative to the Ye9*hfq*/pBAD24Cm. Western blotting of cell lysates was performed with a monoclonal mouse anti-FLAG epitope antibody. Part of the Bio-Rad Stain-Free gel is shown as a loading control. These results are representative of two independent experiments. MW—3-color prestained protein marker.

After exploring the role of Hfq and sRNA RyhB1 in the modulation of Fur expression, we turned our attention to FepA and FecA, the OM transporters/receptors for ferric citrate and ferric enterobactin, respectively. To test whether Hfq might control the expression of *fecA* and *fepA*, *fecA*’-’*gfp* and *fepA*’-’*gfp* translational fusions expressed from the plasmid pFX (pFX-*fecA* and pFX-*fepA*) were constructed and introduced into the wild-type strain Ye9N and Ye9*hfq* (∆*hfq* mutant). In addition, the effect of the complementation or overexpression of Hfq was studied by introducing pBAD-Hfq into Ye9*hfq* and the wild-type strain Ye9N carrying pFX-*fecA* or pFX-*fepA*. All tested strains were grown in LB and in LBD (iron-limiting conditions). As shown in Figure 2A, strain Ye9*hfq* exhibited increased *fecA*’-’*gfp* expression, irrespective of the iron concentration, compared to the wild-type strain (31% in LB and 36% in LBD). Increased expression of the *fepA*’-’*gfp* fusion was also observed in this ∆*hfq* mutant (52% in LB and LBD), independently of the iron concentration (Figure 2A). These results suggest that Hfq may participate in repressing *fecA* and *fepA* expression independently of iron. Interestingly, when the wild-type allele of *hfq* on the plasmid pBAD-Hfq was introduced into Ye9*hfq* and induced, the expression of *fecA* and *fepA* was slightly decreased, but not to wild-type levels.

The effects of *hfq* overexpression from the plasmid pBAD-Hfq in strains Ye9N/pFX-*fecA* and Ye9N/pFX-*fepA* were also analyzed (Figure 2B). Interestingly, an effect of increased Hfq on *fecA*’-’*gfp* was observed in cells grown under iron-limited conditions (LBD medium): fluorescence was slightly decreased (~10%) in comparison with the strain carrying the control vector pBAD24Cm, implying that Hfq inhibits *fecA* expression under these growth conditions. In comparison, the *fepA*’-’*gfp* strain carrying pBAD-Hfq displayed decreased fluorescence relative to the strain carrying pBAD24Cm in stationary-phase cultures grown in both LB and LBD medium (13% and 7%, respectively) (Figure 2B).

These results demonstrate that the lack of *hfq* leads to the upregulation of *fecA* and *fepA* expression irrespective of iron concentration. Therefore, Hfq participates in the repression of both genes. Hfq expressed from a multicopy plasmid had a negative effect on the expression of *fecA* in an iron-depleted culture or independently of iron in the case of *fepA*.

To check whether the Hfq-dependent regulation of *fecA* and *fepA* expression observed using translational fusions is reflected in the level of the encoded proteins, Western blotting analysis was performed (Figure 2C). To facilitate the detection of FecA and FepA, we prepared strains of Ye9N and Ye9*hfq* carrying a chromosomal copy of the desired gene encoding an in-frame translational fusion with a 3×FLAG epitope peptide at the C-terminal end (see Materials and Methods). The empty vector pBAD24Cm or plasmid pBAD-Hfq was then introduced into the FLAG-tagged strains. Western blot analysis with the anti-FLAG antibody was used to detect the FecA-3×FLAG and FepA-3×FLAG proteins in extracts prepared from cells grown to the stationary phase in LBD medium. As expected, significant increases in the levels of FecA-3×FLAG (~100%) and FepA-3×FLAG (~86%) were observed in the ∆*hfq* mutant background (strains Ye9*hfq*FecAflag and Ye9*hfq*FepAflag) in comparison with the parental strain Ye9N (strains Ye9FecAflag and Ye9FepAflag), all carrying the empty plasmid pBAD24Cm. The overexpression of *hfq* from pBAD-Hfq in Ye9*hfq* (complementation assay) decreased the abundance of FecA-3×FLAG (~41%) and FepA-3×FLAG (~35%) compared with the strains carrying pBAD24Cm, which supports the notion that Hfq negatively impacts FecA and FepA abundance under these conditions. It should be noted that we did not see significant changes in the amount of FecA-3×FLAG or FepA-3×FLAG when *hfq* was overexpressed in strains Ye9FecAflag and Ye9FepAflag grown to the stationary phase in LBD medium. Thus, in the wild-type strain Ye9N, additional Hfq provided by the plasmid pBAD-Hfq appeared to result in the decreased expression of the genes encoding the two Fe-siderophore transporters/receptors (*fecA*’-’*gfp* and *fepA*’-’*gfp* expression data), but the production of the proteins showed no significant changes (FecA-3×FLAG and FepA-3×FLAG protein abundance). This discrepancy between the reporter gene and Western blot data could be explained if the effect of Hfq on *fecA* and *fepA* expression detected using the *gfp* fusions is too modest to lead to a measurable decrease in FecA and FepA levels. However, the obtained results confirmed the participation of Hfq in repressing *fecA* and *fepA* expression in *Y. enterocolitica.*

### 2.3. Partial Conservation of the omrA/B Locus in Gammaproteobacteria

**Figure 3:**
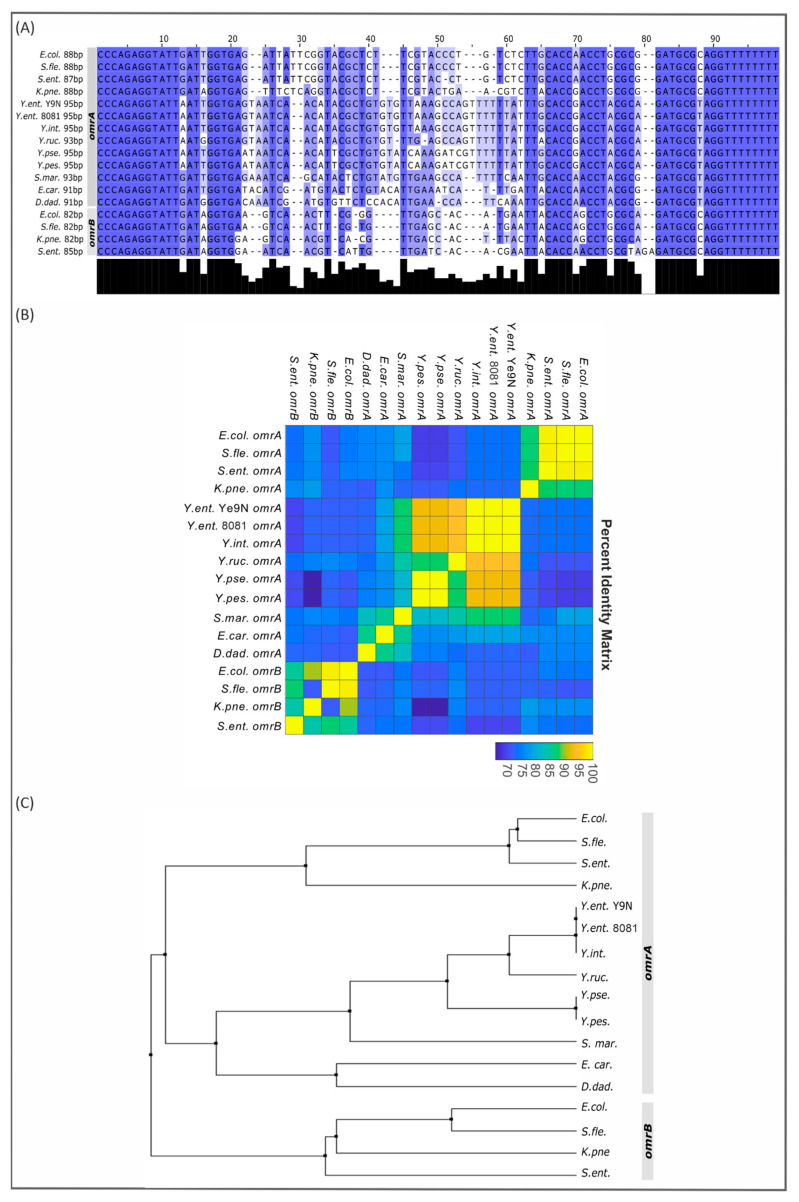
Nucleotide sequence conservation of OmrA and OmrB sRNAs from Gammaproteobacteria. (**A**) Multiple sequence alignment of *omrA* and *omrB* genes from 12 species (13 strains) of Gammaproteobacteria (*Escherichia coli* str. K-12 substr. MG1655 (*E.col.*; NCBI taxid: 511145); *Shigella flexneri* serotype 2a str. 301 (*S.fle.*; NCBI taxid: 198214); *Salmonella enterica* subsp. *enterica* serovar Typhimurium str. 14028S (*S.ent*.; NCBI taxid: 588858); *Klebsiella pneumoniae* subsp. *pneumoniae* HS11286 (*K.pne.*; NCBI taxid: 1125630); *Yersinia enterocolitica* subsp. *palearctica* Ye9N bioserotype 2/O:9 (*Y.ent.* Ye9N; a shotgun genome sequence: Accession number NZ_JAALCX010000053.1); *Yersinia enterocolitica* subsp. *enterocolitica* 8081 (*Y.ent.* 8081; NCBI taxid: 393305); *Yersinia intermedia* (*Y.int*.; NCBI taxid: 631); *Yersinia ruckeri* ATCC 29473 (*Y.ruc*.; NCBI taxid: 527005); *Yersinia pseudotuberculosis* IP 32953 (*Y.pse*.; NCBI taxid: 273123); *Yersinia pestis* str. A1122 (*Y.pes*.; NCBI taxid: 1035377); *Serratia marcescens* strain KS10 (*S.mar*.; NCBI taxid: 615); *Erwinia carotovora* subsp. *atroseptica* SCRI1043 (*E.car*.; NCBI taxid: 218491); and *Dickeya dadantii* 3937 (*D.dad*.; NCBI taxid: 198628)). The alignment was performed using the T-Coffee tool and visualized in Jalview version 2.8.2 [79,80]. The nucleotides are colored by percentage identity; the most highly conserved nucleotides have the darkest color (dark blue signifies more than 80% nucleotide identity; medium blue represents more than 60% identity; light blue corresponds to more than 40% identity, and no color indicates identity below 40%). The level of identity is shown by a histogram at the bottom. The coding region lengths are given. (**B**) Heatmap indicating the percentage identity of *omrA*/*omrB* genes shared by two species. The identity matrix was calculated based on comparisons using the T-Coffee tool. The yellow–blue gradient bar represents the identity percentage scale. (**C**) Phylogenetic tree of *omrA* and *omrB* sequences in Gammaproteobacteria. The diagram was created with Jalview version 2.8.2 [80] based on matrices of average distances for the selected species.

Screens of the *E. coli* genome revealed the existence of two tandemly located sRNA genes, *omrA* and *omrB*, first named *rygA/sraE* and *rygB* [54,55]. The encoded sRNAs OmrA and OmrB are 88 nt and 82 nt in length, respectively, and share nearly identical sequences in their 5′ and 3′ regions [25,55]. Both sRNAs have been shown to downregulate the synthesis of several proteins, including outer membrane receptors for Fe-siderophores, i.e., CirA, FecA and FepA [25,56]. To examine the sequence conservation of OmrA and OmrB among Gammaproteobacteria, 13 genomes were searched for orthologs of these sRNAs: six Yersiniaceae species, namely, human pathogens *Y. enterocolitica* (2 strains of different bioserotypes—Ye9N (bioserotype 2/O:9) and 8081 (bioserotype 1B/O:8)), *Y. pseudotuberculosis* and *Y. pestis*, the fish pathogen *Y. ruckeri*, the environmental species *Y. intermedia* and *Serratia marcescens* (an opportunistic human pathogen), plus 4 Enterobacteriaceae species, *E. coli*, *Shigella flexneri*, *Salmonella enterica* subsp. *Enterica* and *Klebsiella pneumoniae*, and 2 members of the Pectobacteriaceae, the plant pathogens *Erwinia carotovora* and *Dickeya dadantii.* Multiple sequence alignment of the identified *omrA* and *omrB* genes was performed, the pairwise nucleotide identity was calculated, and an average distance tree was created (Figure 3). Interestingly, while both *omrA* and *omrB* genes could be identified in all studied members of the Enterobacteriaceae, only one Omr sRNA gene was recognized in Yersiniaceae and Pectobacteriaceae species. In addition, the Omr sRNAs in the Yersiniaceae are >90 nt in length, which is longer than both OmrA and OmrB in *E. coli.* A high level of sequence conservation was found within the first 21 and last 18 nucleotides of each sRNA, suggesting the importance of these regions for sRNA function. Analysis of the *omr* gene sequences from the selected bacteria provided phylogenetic information on this sRNA regulatory system. The highly conserved Omr sequences of Yersiniaceae are much closer to *E. coli* OmrA than OmrB. *Omr* in *Y. enterocolitica* strain Ye9N shares 93% sequence identity with those in *Y. pseudotuberculosis* and *Y. pestis* and 73% and 68% sequence identity with *E. coli* OmrA and OmrB, respectively. This analysis prompted us to identify the Omr sRNA in strain Ye9N as OmrA.

The genomic context of the *omr* genes in the analyzed Gammaproteobacteria is presented in Table S3. The *omrA* and *omrB* genes in the *E. coli* chromosome are tandemly located within the intergenic region between the genes *aas*, encoding 2-acylglycerophospho-ethanolamine acyl transferase/acyl-acyl carrier protein synthetase, and *galR*, encoding a transcriptional regulator. The same upstream/downstream genes flanking the *omrA* and *omrB* sRNA genes are present in the other Enterobacteriaceae species/strains examined (*S. flexneri* 301, *S. enterica* sv Typhimurium 14028S and *K. pneumoniae* subsp. *pneumoniae* HS11286). Analysis of the genome sequence of *Y. enterocolitica* strain Ye9N (bioserotype 2/O:9) localized the single *omrA* gene between the genes *tnp*, encoding IS110 family transposase, and *aas*. The *aas* and *omrA* genes are in the same transcriptional orientation, whereas *tnp* is oriented divergently. The same genomic organization is present in *Y. enterocolitica* strain 8081 of bioserotype 1B/O:8. Interestingly, the genomic context of the *omrA* gene varies in other Yersiniaceae species/strains. In *Y. pseudotuberculosis* IP 32953, *Y. pestis* A1122, *Y. intermedia* and *S. marcescens* KS10, *omrA* is located between the genes *bisC*, encoding the molybdopterin guanine dinucleotide-containing S/N-oxide reductase, and *aas*, while the genes flanking *omrA* in *Y. ruckeri* ATCC 29473 are *aas* and *hp*, encoding a hypothetical protein. In *E. carotovora subsp. atroseptica* SCRI1043, the *omrA* gene is located between *aas* and a gene encoding a methyl-accepting chemotaxis protein, while in *D. dadantii* 3937, the neighboring genes of *omrA* are quite different to those in all the other analyzed species: it is located between *bglG*, encoding the beta-glucoside *bgl* operon antiterminator, and *lpxO*, encoding Fe^2+^/alpha-ketoglutarate-dependent dioxygenase LpxO. In summary, this analysis revealed that the genomic context is highly conserved when both *omrA* and *omrB* are present but is only partially conserved when there is just one *omr* gene.

### 2.4. Y. enterocolitica’s OmrA sRNA Decreases fecA and fepA Levels

**Figure 4:**
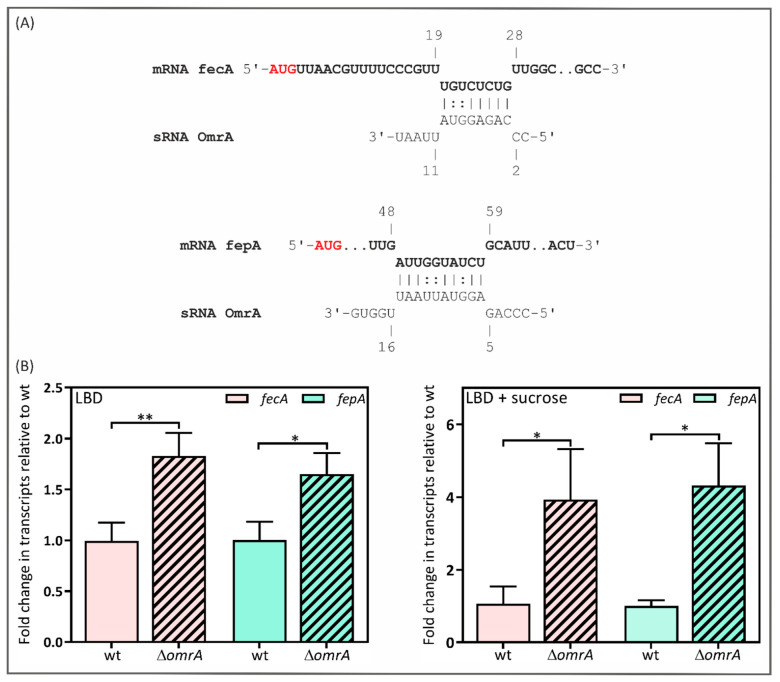
The *Y. enterocolitica fecA* and *fepA* genes are negative regulatory targets of sRNA OmrA. (**A**) Predicted base pairing between sRNA OmrA and the *fecA* and *fepA* transcripts. The mRNA sequences are in bold, with the translation start codons marked in red. Nucleotides are numbered from the first base of the start codon. (**B**) The levels of *fecA* and *fepA* transcripts assessed by RT-qPCR in Ye9N (wt) and Ye9*omrA* (∆*omrA*). The analysis was performed using RNA prepared from cells grown to the early stationary phase in LBD (**left panel**) and LBD supplemented with 20% sucrose (**right panel**). Relative *fecA* and *fepA* transcript levels, normalized to the amount of 16S rRNA, are shown, taking the mRNA level in Ye9N as 1. The presented data are the mean values (±standard deviation) obtained from at least three independent experiments. Significance was calculated using Student’s unpaired *t*-test (** *p* < 0.01, * *p* < 0.05).

If OmrA from *Y. enterocolitica* directly regulates *fecA* or *fepA* transcript levels, we would expect to see base pairing between OmrA and the respective mRNAs. Using the software IntaRNA version 5.0.7, we were able to predict base pairing between OmrA and both analyzed mRNAs (Figure 4A). This analysis revealed complementarity between positions +20 and +27 nt of the *fecA* mRNA and between +49 and +58 nt of the *fepA* mRNA, downstream of the translation start site. The *fecA* mRNA region is complementary to nucleotides 3 to 10 of OmrA, while the *fepA* mRNA region is complementary to nucleotides 6 to 15 of OmrA. Interestingly, potential pairing with OmrA occurs within the early coding sequences of the *fecA* and *fepA* mRNAs instead of the 5′ UTR. To reveal whether this sRNA controls *fecA* and *fepA* expression, we measured the effect of *omrA* deletion on *fecA* and *fepA* transcript levels by RT-qPCR. An isogenic ∆*omrA* mutant was generated in *Y. enterocolitica* strain Ye9N (strain Ye9*omrA*) by marked allelic exchange using a construct based on the *sacB*-dependent suicide plasmid pDS132 (see Materials and Methods). This null mutation prevented the production of the OmrA mRNA (Figure S1). RT-qPCR analysis was performed using RNA isolated from the wild-type *Y. enterocolitica* strain Ye9N and the *omrA* null mutant grown in LBD and in LBD supplemented with 20% sucrose, known to induce *omrA* expression in *E. coli* [24]. As shown in Figure 4B, the deletion of *omrA* resulted in increased levels of the *fecA* and *fepA* transcripts (*fecA* displayed a 1.8-fold increase, and *fepA* increased 1.7-fold). More visible differences were observed when strains were grown in the iron-limited medium supplemented with sucrose (3.9-fold for *fecA* and 4.3-fold for *fepA*). The nature of the molecular interactions between OmrA and these mRNAs in *Y. enterocolitica* has yet to be determined and requires further study.

### 2.5. Influence of OmpR and Environmental Conditions on omrA Promoter Function

**Figure 5:**
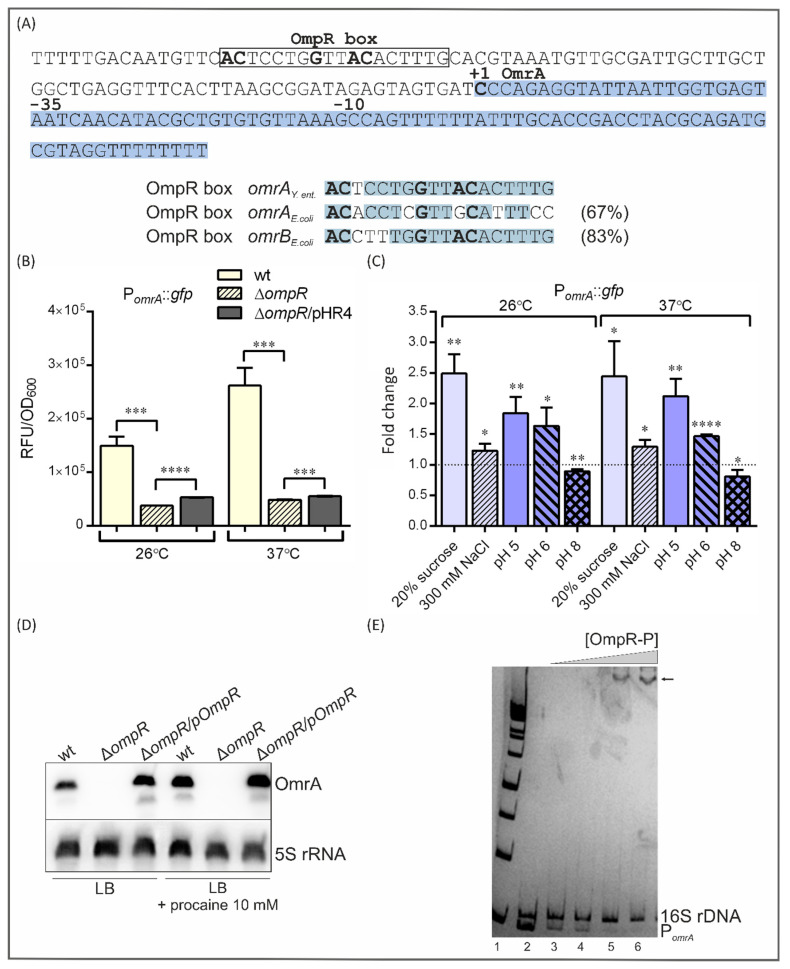
Influence of OmpR on *omrA* promoter function. (**A**) Putative OmpR-binding site (OmpR box) identified in the *omrA* regulatory region of *Y. enterocolitica* strain Ye9N (boxed). The 5′ end of the *omrA* transcript is shaded blue. Below, the OmpR boxes identified in the promoters of *omrA* and *omrB* in *E. coli* [24] are aligned with the OmpR box recognized in the Ye9N *omrA* promoter (% identity is shown). Nucleotides crucial for OmpR interaction are shown in bold. Identical nucleotides are shaded gray. (**B**) Analysis of *omrA* expression in the wild type (Ye9N), ∆*ompR* mutant (AR4) and strain AR4 complemented with pHR4, using a P*_omrA_*::*gfp* transcriptional fusion. Strains were cultivated in LB at 26 °C or 37 °C to stationary phase. The data represent mean fluorescence activity values normalized to the OD_600_ of the culture (±standard deviation) from a representative experiment performed using at least triplicate cultures of each strain. Significance was calculated using Student’s unpaired *t*-test (**** *p* < 0.0001, *** *p* < 0.001). (**C**) Fluorescence intensity of the wild-type strain Ye9N harboring the P*_omrA_*::*gfp* fusion plasmid grown in LB to exponential phase then exposed to high osmolarity or different pH conditions for 1 h at 26 °C or 37 °C. Fold change relative to strain Ye9N grown in LB is shown. Significance was calculated using Student’s unpaired *t*-test (**** *p* < 0.0001, ** *p* < 0.01, * *p* < 0.05). (**D**) Northern blot showing the levels of *omrA* mRNA in the wild-type strain Ye9N, ∆*ompR* mutant (strain AR4) and complemented strain AR4/pOmpR grown to exponential phase in LB with or without the addition of procaine (10 mM). As a loading control, the level of 5S rRNA was examined. (**E**) EMSA analysis to study the ability of His-tagged OmpR-P to bind to the promoter region of *omrA* in vitro. The 250 bp *omrA* promoter fragment was incubated without protein (lane 2) or with 12.4 μM (lane 3), 24.9 μM (lane 4), 37.3 μM (lane 5) or 49.7 μM (lane 6) His-tagged OmpR-P. Lane 1, MassRuler DNA Ladder Low Range. A 304 bp 16S rDNA fragment was included in the reaction mixtures as a competitor and negative control. Unbound DNA and protein/DNA complexes are indicated.

Previous studies in *E. coli* have identified the OmpR protein as a positive regulator of sRNA OmrA and OmrB transcription [24,60]. An examination of the *Y. enterocolitica* Ye9N *omrA* promoter sequence identified one putative OmpR-binding site (OmpR box) exhibiting 67% and 83% identity to the OmpR boxes found in the *E. coli omrA* and *omrB* promoter regions, respectively (Figure 5A). These OmpR boxes were recognized based on their similarity to OmpR-binding motifs found in the regulatory regions of the *E. coli ompC* and *ompF* porin genes [24,81]. The conserved motif GxxxC and AC base pairs are critical for OmpR binding [81,82,83]. The OmpR box in the Ye9N *omrA* gene promoter is located at −82 to −63 nt relative to the transcription initiation site (+1). The presence of a putative OmpR-binding motif in the Ye9N *omrA* regulatory sequence prompted us to examine the role of OmpR in the regulation of *omrA* transcription by creating a fusion of the *omrA* promoter with *gfp* in the plasmid vector pPROBE-TT’ (see Materials and Methods). This plasmid, pPOmrA, was introduced into the wild-type strain Ye9N and the ∆*ompR* mutant AR4 (constructed using a reverse genetic PCR-based strategy [84]). Complementation analysis was performed by transforming strain AR4/pPOmrA with the plasmid pHR4 expressing the active OmpR protein [84]. Cultures of strains carrying pPOmrA were grown to the stationary phase in LB medium at 26 °C and 37 °C, and green fluorescence was measured (Figure 5B).

The data presented in Figure 5B show that when grown at 26 °C or 37 °C, the ∆*ompR* mutant AR4 displayed a significant decrease (4-fold and 5.4-fold, respectively) in reporter gene expression compared to the parental strain Ye9N, suggesting that OmpR is involved in the positive regulation of the *omrA* gene. Complementation of the *ompR* mutation by OmpR expressed *in trans* (strain AR4/pPOmrA/pHR4) caused a slight increase in fluorescence compared to the ∆*ompR* mutant strain at both studied temperatures. The link between OmrA and OmpR might be complex, with the possible existence of a negative regulatory circuit whereby OmrA can limit *ompR* expression through feedback control (see 2.6). We cannot rule out the possibility that an optimal concentration of phosphorylated OmpR is required to control the amount of OmrA. Moreover, in the complementation assay, there will be an excess of OmpR relative to EnvZ, and this imbalance may influence the expression of *omrA*.

The activity of the *omrA* promoter in Ye9N was higher at 37 °C than at 26 °C (1.75-fold), indicating that the transcription of this gene is subject to temperature regulation. This thermoregulation was also observed in strain AR4, indicating that OmpR plays no part in the response of *omrA* to changes in temperature.

The EnvZ/OmpR TCS modulates the expression of several genes in *E. coli* and *Salmonella* in response to altered osmolarity and pH [62,63]. These environmental signals change the DNA-binding affinity of the transcriptional activator protein OmpR. Therefore, we next examined the influence of these environmental cues on the activity of the *omrA* promoter (Figure 5C). First, *omrA* promoter activity was assessed in strain Ye9N (Ye9N/pPOmrA) grown in LB medium to the exponential phase and then incubated for 1 h at 26 °C or 37 °C in LB supplemented with 20% sucrose or 0.3 M NaCl (Figure 5C). These high-osmolarity conditions caused increases in the activity of the *omrA* promoter of 2.5-fold in 20% sucrose and 1.2-fold in 0.3 M NaCl irrespective of the temperature. To determine whether the pH of the growth medium influences the expression of *omrA*, cultures were grown in LB medium to the exponential phase and then incubated for 1 h at 26 °C or 37 °C in LB medium at pH 5, 6 or 8. Acid pH significantly increased the activity of the P*_omrA_*::*gfp* transcriptional fusion, while this activity was significantly decreased at pH 8.0 (Figure 5C). No effect of temperature was observed. Procaine, a local anesthetic, is also known to activate the OmpR regulator [85,86]. Therefore, we examined the influence of procaine on the regulation of *omrA* expression by Northern blotting performed using RNA prepared from Ye9N and AR4 (∆*ompR*) cells grown to the exponential phase in the presence and absence of this compound. As shown in Figure 5D, the level of the *omrA* transcript was increased in strain Ye9N after the addition of 10 mM procaine. In strain AR4 (∆*ompR*), no *omrA* transcripts could be detected in cells grown in LB with or without procaine, confirming the importance of OmpR as an activator of *omrA* transcription in the exponential phase. Interestingly, as shown in Figure 5B, stationary-phase cells of the ∆*ompR* mutant displayed a significant decrease in reporter gene expression compared to the parental strain, although a residual level of *omrA* expression was still detected.

In summary, these results reveal the role of OmpR in the activation of *omrA* expression and imply that OmpR may directly influence *omrA* transcription, i.e., by binding to the promoter region of *omrA*. One potential OmpR-binding site was recognized in the *omrA* promoter region of strain Ye9N (Figure 5A). To determine whether this OmpR box functions as a binding site for OmpR in vitro, we performed an electrophoretic mobility shift assay (EMSA) using a 250 bp fragment encompassing the *omrA* regulatory region and purified OmpR-His6 protein [74], phosphorylated in vitro by acetyl-*p*. The DNA fragment was incubated with increasing amounts of the His-tagged OmpR-P protein. As a control, a 304 bp 16S rDNA fragment was included in the binding reactions. As shown in Figure 5E, slower-migrating OmpR-P/DNA complexes were observed in the presence of OmpR-P at concentrations of 37.3 µM and above. OmpR-P failed to interact with the 16S rDNA fragment used as a negative control. These results demonstrate that the regulation of *omrA* transcription by OmpR is mediated by the direct binding of this regulatory protein to the *omrA* promoter.

### 2.6. OmrA Regulates ompR Expression Post-Transcriptionally

**Figure 6:**
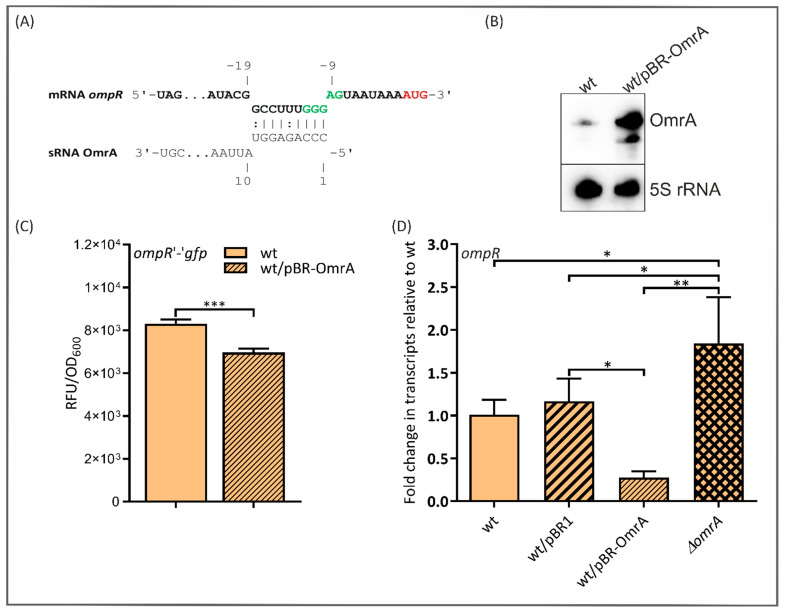
OmrA inhibits *ompR* expression at the post-transcriptional level. (**A**) Predicted base pairing between sRNA OmrA and the *ompR* mRNA of *Y. enterocolitica* Ye9N. mRNA sequences are shown in bold. The AUG translation start codon and putative Shine–Dalgarno sequences are in bold and marked in red and green, respectively. (**B**) Northern blot showing the level of *omrA* mRNA in the wild-type strain Ye9N and Ye9N carrying pBR-OmrA. Strains were grown in LB to stationary phase. As a loading control, the level of 5S rRNA was examined. (**C**) Analysis of *ompR* expression in strain Ye9N carrying the *ompR*’-’*gfp* translational fusion (pFX-*ompR*) after growth in LB to stationary phase in the presence or absence of plasmid pBR-OmrA. The data represent mean fluorescence activity values normalized to the OD_600_ of the culture (±standard deviation) from at least three independent experiments, each with three separate cultures per strain. Significance was calculated using Student’s unpaired *t*-test (*** *p* < 0.001). (**D**) The level of *ompR* transcripts assessed by RT-qPCR in Ye9N, Ye9N/pBR1, Ye9N/pBR-OmrA and the Δ*omrA* mutant. The analysis was performed using RNA prepared from cells grown to early stationary phase in LB + 20% sucrose. Relative *ompR* transcript levels, normalized to the amount of 16S rRNA, are shown, taking the mRNA level in Ye9N as 1. The presented data are the mean values (±standard deviation) obtained from at least three independent experiments. Significance was calculated using one-way ANOVA (** *p* < 0.01, * *p* < 0.05).

Since two redundant *E. coli* sRNAs, OmrA and OmrB, downregulate the expression of the OmpR transcriptional regulator [24], we investigated the potential OmrA-mediated regulation of the *ompR* gene in *Y. enterocolitica.* The IntaRNA program yielded a short possible predicted pairing between OmrA and the Ye9N *ompR* mRNA near the RBS and translation initiation codon (Figure 6A). This involves the conserved 5′ end of OmrA and nts −10 to −18 of the *ompR* mRNA relative to AUG. The site of interaction of OmrA with *ompR* in *Y. enterocolitica* is extremely similar to the one reported in *E. coli* [56]. To test whether sRNA OmrA is involved in the regulation of *ompR* expression in *Y. enterocolitica*, the plasmid pFX-*ompR* (a kind gift from Prof. O. Rossier) [45] carrying an *ompR*’-’*gfp* translational fusion was applied. Moreover, the plasmid pBR-OmrA was created by cloning the Ye9N *omrA* gene into the expression vector pBR-plac [24], as in the case of sRNA *ryhB1*. The *omrA* transcript abundance was assessed in cells grown to the stationary phase in LB by Northern blotting. As shown in Figure 6B, the *omrA* mRNA (transcript of the chromosomal gene) was barely detectable in strain Ye9N but was strikingly more abundant in Ye9N carrying the plasmid pBR-OmrA. This demonstrated that ectopically expressed OmrA could be used to study the post-transcriptional regulation of *ompR* expression. As shown in Figure 6C, strain Ye9N carrying the *ompR*’-’*gfp* fusion with an enhanced level of OmrA, grown to the stationary phase, displayed a ~24% decrease in fluorescence intensity compared to the strain without the plasmid pBR-OmrA. Since the effect of OmrA on the *ompR*’- ’*gfp* fusion activity was quite modest, we used RT-qPCR to estimate the level of the *ompR* transcript in strains with different OmrA contents (Figure 6D). This analysis showed that the plasmid-borne OmrA significantly reduced *ompR* expression (3.6-fold). Moreover, the deletion of *omrA* resulted in an increased level of the *ompR* transcript. These data support the notion that OmrA represses the expression of *ompR* in *Y*. *enterocolitica*.

### 2.7. Requirement for OmpR in the OmrA-Dependent Regulation of fecA and fepA Expression

**Figure 7:**
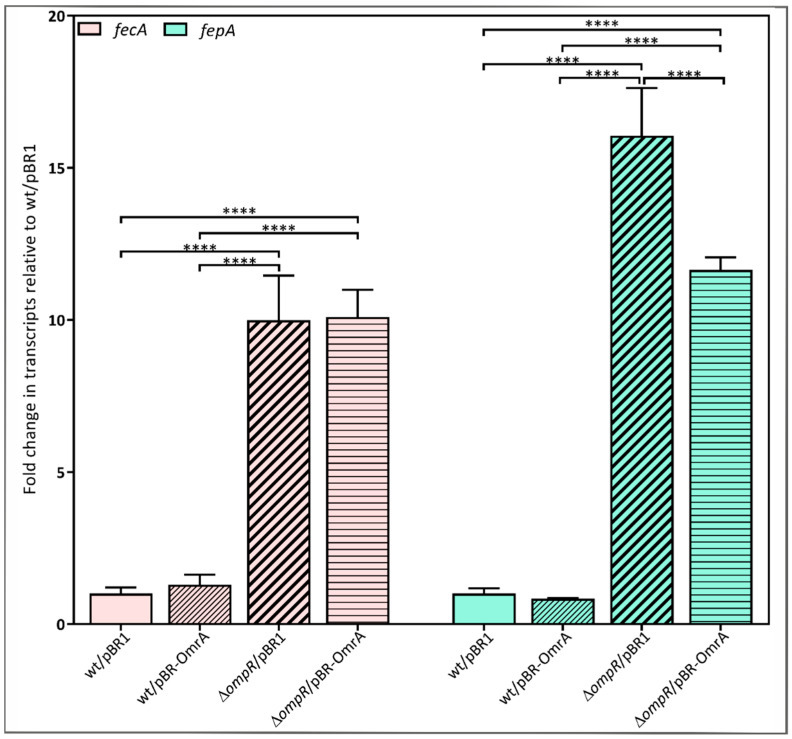
Levels of *fecA* and *fepA* transcripts in the wild-type (Ye9N) and ∆*ompR* strains (AR4) transformed with pBR-OmrA or the empty vector pBR1. The abundance of *fecA* and *fepA* mRNAs was assessed by RT-qPCR after the growth of these strains in LBD. Relative *fecA* and *fepA* transcript levels, normalized to the amount of 16S rRNA, are shown, taking the mRNA level in Ye9N/pBR1 as 1. The presented data are the mean values (±standard deviation) from at least two independent experiments. Significance was calculated using one-way ANOVA (**** *p* < 0.0001).

Following the demonstration that OmpR induces *omrA* transcription in *Y. enterocolitica* and there is negative feedback between sRNA OmrA and *ompR* expression, we decided to examine the role of OmrA in the post-transcriptional regulation of *fecA* and *fepA* in the *ompR*-null mutant background. The *fecA* and *fepA* transcripts were quantified in strain AR4 (∆*ompR*) and compared with the wild-type Ye9N, both carrying plasmid-borne *omrA* (pBR-OmrA) or the empty vector (pBR1). The results presented in Figure 7 reveal an increase in the levels of the *fecA* and *fepA* transcripts in the Δ*ompR* strain AR4/pBR1 relative to the wild-type strain Ye9N/pBR1 (10-fold and 16-fold), confirming previous data showing that OmpR directly inhibits *fecA* and *fepA* transcription in *Y. enterocolitica* [22]. In light of our findings regarding OmrA, this effect may also result from the lack of this sRNA, since it is not expressed in the Δ*ompR* mutant background. Interestingly, no effect of ectopically expressed OmrA on the *fecA* and *fepA* mRNA levels was observed in the wild-type strain, suggesting that a combination of transcriptional and post-transcriptional regulatory mechanisms involving OmrA and OmpR could inversely modulate the abundance of both transcripts. Surprisingly, only a moderate reduction in *fepA* mRNA levels (27%) was observed in the ∆*ompR* mutant background in the presence of plasmid-borne *omrA*, while the level of *fecA* transcripts remained unchanged. These results suggest that *fepA* expression, in contrast to *fecA*, may be silenced by OmrA independently of OmpR. Since the inhibitory effect on *fepA* mRNA levels in the presence of OmrA is modest, it is possible that the abundance/activity of other factors (Hfq, RNase E), known to be involved in OmrA-dependent regulation, may be decreased in the ∆*ompR* mutant background.

## 3. Discussion

In this study, we investigated whether the RNA chaperone Hfq influences the expression of the gene encoding Fur, the master regulator of iron homeostasis in *Y. enterocolitica*, and two genes belonging to the Fur regulon, *fecA* and *fepA*, encoding OM receptors/transporters for ferric citrate and ferric enterobactin, respectively. Two experimental approaches were employed: (i) measuring fluorescence produced by translational fusions of *fur*, *fecA* and *fepA* with *gfp* and (ii) monitoring the production of FLAG-tagged Fur, FepA and FecA proteins by immunoblotting.

The study of translational fusions of *fur* with *gfp* demonstrated that the absence of chromosomally encoded Hfq caused the upregulation of *fur* expression. Hfq-dependent silencing of *fur* expression was observed independently of the presence of iron ions. However, in wild-type cells, the overexpression of Hfq from a plasmid led to the downregulation of *fur* expression under iron-starved conditions, while upregulation was observed in the presence of iron. These iron-dependent alterations in *fur* expression seen in the presence of plasmid-borne Hfq suggest that the link between iron, Fur and Hfq might be complex. Western blot analysis confirmed the inhibitory effect of Hfq on the production of FLAG-tagged Fur in *Y. enterocolitica*. However, this polypeptide was clearly produced under iron-limited conditions, which suggests that Hfq-dependent production of Fur occurs in an iron-responsive manner. Hfq usually acts in conjunction with sRNAs to modulate mRNA stability and/or translation [39]. Therefore, we hypothesized the involvement of putative regulatory sRNAs, whose activity is triggered by iron starvation and which interact with Hfq to decrease Fur abundance.

Hfq-dependent sRNA RyhB, whose expression is negatively regulated by iron (Fe^2+^-Fur), may play a role in the post-transcriptional control of *fur* in *E. coli* [11,35]. The analysis of the *Y. enterocolitica* genome sequence revealed the presence of two RyhB homologs, named RyhB1 and RyhB2. The potential base pairing of RyhB1/RyhB2 with a single site in the coding sequence of the *fur* mRNA was recognized. The initial characterization of *Y. enterocolitica* RyhB1 revealed its role in the negative regulation of *fur* expression. The influence of RyhB2 on *Y. enterocolitica fur* has yet to be determined. Interestingly, reciprocal regulation between Fur and two RyhB homologs in *Y. pestis* has recently been demonstrated [87].

To clarify whether Hfq participates in the post-transcriptional regulation of the *fecA* and *fepA* genes, expression studies were performed under iron-replete and iron-limiting conditions (release from Fe^+2^-Fur repressor activity). A comparative analysis of strains with different Hfq contents, using plasmid-encoded translational fusions of *fecA* and *fepA* with a *gfp* reporter gene, indicated that Hfq represses the expression of these genes irrespective of iron. Interestingly, the silencing of *fecA* and *fepA* by overexpressed Hfq in iron-starved conditions was moderate, suggesting that an optimal concentration of Hfq is required to control the amounts of the transporters FecA and FepA. It was previously found that raising Hfq levels modestly increases the accumulation of sRNAs and hence the silencing of some target genes [88,89]. Finally, an analysis of the abundance of FLAG-tagged FecA and FepA expressed from their chromosomal loci under iron-depleted conditions confirmed that Hfq negatively affects the production of these proteins. The demonstration that Hfq regulates the expression of these ferric siderophore transporters is in line with the findings of a previous study showing that, in *Y. enterocolitica* bioserotype 1B/O:8 strains, Hfq inhibits the expression of the OM receptor FyuA for the uptake of Fe-yersiniabactin (Fe-Ybt), as well as the synthesis of Ybt [44]. The genes necessary for Ybt synthesis, uptake and regulation are localized within a High-Pathogenicity Island (HPI) that is not present in *Y. enterocolitica* strains of biotypes 2–5, which include strain Ye9N, the object of our research [18,90].

Our finding that Hfq affects the expression of proteins involved in iron acquisition and the master regulator Fur indicates that this RNA chaperone plays a key role in iron homeostasis in *Y. enterocolitica*. It is also worth adding that the expression of several OM proteins involved in the pathogenicity of *Y. enterocolitica* 1B/O:8 that are not regulated by iron is also regulated by Hfq [45]. It appears that Hfq is involved in both iron-dependent and -independent gene regulation in *Y. enterocolitica*, as is the case in *E. coli* [91] and *Neisseria meningitidis* [92]. Thus, we cannot exclude the possibility that Hfq may play both direct and indirect roles in the post-transcriptional regulation of the *fecA* and *fepA* genes in *Y. enterocolitica*.

Previously, Guillier and Gottesman [24,56] showed that the homologous sRNAs OmrA and OmrB in *E. coli*, bound to Hfq, exert post-transcriptional control over the expression of the genes *cirA*, *fecA* and *fepA*, encoding OM transporters for ferric siderophores, by pairing with and altering the stability of their mRNAs. Interestingly, while two redundant genes, *omrA* and *omrB*, are present in *E. coli*, only *omrA* was found in the genomes of the pathogenic *Yersinia* species *Y. enterocolitica*, *Y. pestis* and *Y. pseudotuberculosis*. Overall, the OmrA sequence is conserved in the genus *Yersinia*, but there are some differences in the core region compared with OmrA in *E. coli*. In light of the finding that Hfq can repress the expression of *fecA* and *fepA* in *Y. enterocolitica*, we sought to determine whether the sRNA OmrA participates in this regulation. It was also interesting because the homologous sRNAs might exhibit variations in their function that reflect the lifestyle of the bacteria concerned [93]. Notably, the sRNA OmrA in *E. coli* has been identified as a negative regulator of motility, which contrasts with the positive role it plays in *Erwinia amylovora* [94].

To confirm the participation of OmrA in the post-transcriptional inhibition of *fecA* and *fepA* expression, RT-qPCR analysis was performed to examine levels of the gene transcripts in the parental strain and a ∆*omrA* mutant. The results suggest that both analyzed genes are repressed by OmrA in *Y. enterocolitica*, especially in a medium supplemented with sucrose. These findings are in agreement with those of studies in *E. coli*, although *fecA* was shown to be a negative target for both OmrA and OmrB in this species, whereas only OmrA affected *fepA* expression [24].

Direct negative regulation by an Hfq-bound sRNA is usually dependent on base pairing between the sRNA and its target mRNA near the ribosome-binding site [37]. Potential base pairing between the conserved 5′ region of *Y. enterocolitica* OmrA and the *fepA* and *fecA* mRNAs was identified in their coding sequences. However, the importance of this predicted base pairing in silencing *fecA* and *fepA* requires experimental verification. This is particularly important in light of results concerning the pairing of the *fepA* transcript with sRNAs OmrA/B in *E. coli*. It was shown that OmrA/B represses *fepA* expression by targeting a stem-loop structure within the mRNA coding sequence at position +19, which seems to be necessary for the activation of translation initiation [95]. Our bioinformatic analysis showed that the *fepA* mRNA sequence in *Y. enterocolitica* differs considerably from that in *E. coli* (contains deletions and subtle single or multiple base variations), although a stem-loop-like structure is present at position +12 of the *fepA* coding sequence *(*Figure S2A). Moreover, OmrA may form a duplex with this predicted structure (Figure S2B). If, similarly to *E. coli*, this stem-loop region activates the translation of *fepA*, OmrA might repress *fepA* expression by targeting this structure. Notably, an analogous secondary structure within the *fecA* mRNA was not recognized using UNAFold software version 3.5. Further investigation of the *Y*. *enterocolitica* OmrA/*fepA* mRNA interaction is clearly required.

The present study revealed that *omrA* expression in *Y. enterocolitica* is induced in response to environmental signals such as high temperature and osmolarity or low pH, which suggests that the synthesis of OmrA is tightly regulated and restricted to highly specific conditions, like sRNAs in general [96]. While the mechanism of thermoregulation of *omrA* in *Y. enterocolitica* has yet to be characterized, our findings suggest that the EnvZ/OmpR TCS is responsible for regulating *omrA* expression in response to high osmolarity, low pH or the presence of procaine, all factors known to activate the EnvZ/OmpR TCS in *E. coli* [61,62,85,86,97]. By applying a P*_omrA_*::*gfp* transcriptional fusion and Northern blotting, we demonstrated that OmpR strongly and positively affects *omrA* expression. The in vitro binding of *Y. enterocolitica* OmpR to an *omrA* promoter region fragment, including a putative OmpR-binding site identified in silico, was confirmed. These findings demonstrate the direct involvement of OmpR in the positive regulation of sRNA OmrA in *Y. enterocolitica.* It should be noted that although the nucleotide sequence of *Y. enterocolitica omrA* exhibits higher similarity to *E. coli omrA* than to *omrB* (73% vs. 68%), the regulation of *Y. enterocolitica omrA* expression most closely resembles that observed for *E. coli omrB* [24,60].

We also found that the *ompR* mRNA may be a target for silencing by OmrA in *Y. enterocolitica*. Complementarity between the conserved 5′ end of OmrA and a region upstream of the start codon in the *ompR* mRNA, defined in silico, is very similar to that observed in *E. coli* and appears to be sufficient to downregulate *ompR* expression. Our results show that the transcription of *omrA* in *Y. enterocolitica* is activated by OmpR (probably phosphorylated OmpR), and in turn, OmrA represses the synthesis of OmpR. This is equivalent to the regulatory loop described for *E. coli* [24,56]. Given the existence of such a regulatory circuit, we performed RT-qPCR analysis to estimate the role of OmrA in the post-transcriptional regulation of *fecA* and *fepA* expression in the *ompR* mutant background. The absence of OmpR increased the abundance of both transcripts, confirming our previous finding that the expression of these ferric siderophore transporters is controlled at the level of transcription by the regulator OmpR [22]. The results of this study indicate that this effect may also be due to the lack of OmrA in the *ompR* mutant. Interestingly, we observed no significant effect of plasmid-borne OmrA on the levels of the *fecA* and *fepA* mRNAs in the wild-type strain, which might result from an uncharacterized regulatory mechanism operating in *Y. enterocolitica*. This unexpected finding may in part be due to the inhibitory effect of plasmid-borne OmrA on the level of OmpR, being the repressor of *fecA* and *fepA* transcription, resulting in the increased expression of both. On the other hand, OmrA may directly target both mRNAs, leading to their silencing. Interestingly, in the strain lacking OmpR, no effect of OmrA on *fecA* mRNA abundance was observed, and a moderate OmrA-dependent reduction in *fepA* transcript levels occurred. We assume that the absence of OmpR might decrease the level of other regulatory factors, resulting in the impairment of OmrA activity. It was previously shown that the activity of sRNAs and hence the stability of mRNAs may depend on two factors: RNase E and Hfq [35,56]. Nothing is currently known about the influence of OmpR on Hfq and RNase E expression in *Y. enterocolitica.* However, OmpR was found to induce Hfq expression in *Salmonella enterica* sv. Typhi [98].

**Figure 8:**
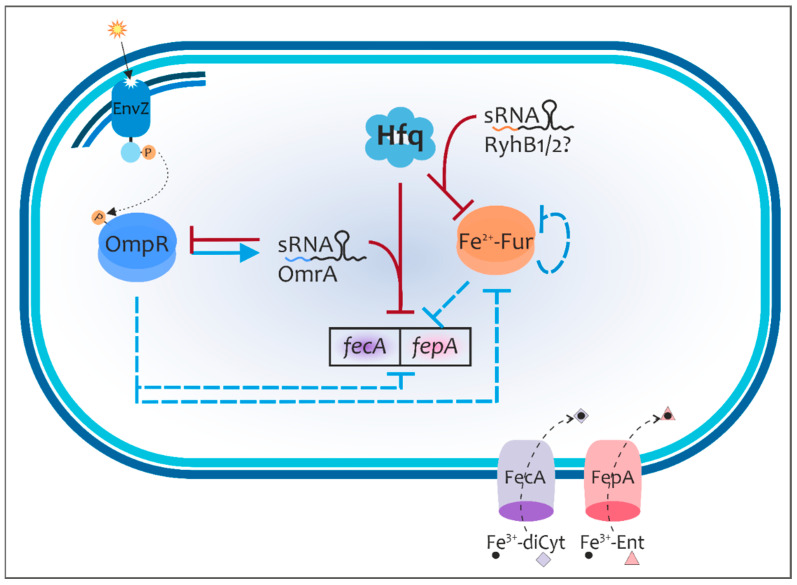
Model for the interplay between regulatory proteins OmpR, Fur, Hfq and sRNAs RyhB1 and OmrA in the regulation of iron acquisition in *Y*. *enterocolitica*. The production of FecA and FepA, the OM transporters for ferric citrate and ferric enterobactin, is controlled by OmpR via direct regulation of the transcription of the *fecA* and *fepA* genes, plus *fur*, encoding the Fur protein, the major transcriptional repressor of *fecA* and *fepA* [22]. We hypothesize that the RNA chaperone Hfq acts in concert with different sRNAs to exert its post-transcriptional effects on the Fur repressor and transporters FecA and FepA. Hfq silences the expression of *fur* at the post-transcriptional level in stationary-phase cells grown in iron-depleted medium, with the participation of putative cofactors (sRNAs RyhB1/RyhB2?). Production of FecA and FepA is negatively controlled by Hfq at the post-transcriptional level. The silencing of *fecA* and *fepA* depends on the sRNA OmrA, and Hfq is important in this regulation. Finally, the production of the sRNA OmrA is controlled by OmpR through its direct positive effect on *omrA* transcription. In turn, OmrA post-transcriptionally controls the expression of OmpR; thus, a coherent feed-forward regulatory loop exists. Dashed blue lines—previously reported regulation of transcription; red and blue lines—regulatory pathways identified in this study (post-transcriptional and transcriptional regulation, respectively). Lines ending with a perpendicular bar indicate a negative interaction; lines ending with an arrowhead indicate a positive effect.

Taken together, the results of this study indicate that the RNA chaperone Hfq and sRNA OmrA, and presumably RyhB homologs, are required in concert with the regulator OmpR for fine-tuning iron homeostasis in *Y. enterocolitica* by modulating the expression of the Fur regulator and iron uptake transporters for ferric citrate (FecA) and ferric enterobactin (FepA). Our previous studies on *Y. enterocolitica* have demonstrated that the expression of the regulator Fur and both transporters is controlled at the level of transcription by OmpR [22]. The present study has uncovered an additional layer of control for a number of these OmpR targets. We propose a model in which several regulatory factors influence the expression of *fur*, *fecA* and *fepA* at the transcriptional and post-transcriptional levels (Figure 8).

## 4. Materials and Methods

### 4.1. Strains, Plasmids, Media and Growth Conditions

The *Y. enterocolitica* and *E. coli* strains and plasmids used in this work are described in Table S1. *Y. enterocolitica* strain Ye9N of bioserotype 2/O:9 and its derivatives were used throughout. *E. coli* strains S17-1 λpir [99], CC118 λpir [100], TOP 10F′ (Thermo Fisher Scientific, Waltham, MA, USA), DH5α [101] and BL21(DE3) were used as hosts for recombinant plasmids. Unless otherwise indicated, *Y. enterocolitica* strains were routinely grown at 26 °C in LB broth (Lennox, BioShop, Burlington, ON, Canada) or on LB agar. When necessary, LB was supplemented with the appropriate antibiotics at the following concentrations: 25 μg/mL chloramphenicol (Cm, 20 µg/mL for *E. coli*), 40 µg/mL gentamicin (Gm; 10 µg/mL for *E. coli*), 50 µg/mL kanamycin (Km), 30 µg/mL nalidixic acid (Nal), 12.5 µg/mL tetracycline (Tet), 50 µg/mL trimethoprim (Tp) and 100 µg/mL spectinomycin (Sp). Iron depletion was achieved by growth in LB supplemented with an iron chelator (150 µM 2,2-dipyridyl; LBD). Hfq expression from pBAD-Hfq was induced by L-arabinose (2%) [101]. The effects of high osmolarity (20% sucrose or 0.3 M NaCl), different pH (pH 5.0, 6.0 and 8.0) and procaine (10 mM) were studied. Cells were grown overnight (to OD_600_ ~1.5), diluted to OD_600_ 0.1 and grown for ~4 h until OD_600_ 0.4 when the required compounds were added. To investigate the effect of acidic and alkaline conditions, the pH of LB medium was adjusted to 5.0 and 6.0 with MES buffer and 8.0 with MOPS buffer. Untreated cells were cultured in parallel as a control.

### 4.2. Molecular Biology Techniques

Standard DNA manipulation methods, i.e., polymerase chain reactions (PCRs), restriction digests, ligations and DNA electrophoresis, were performed as described previously [102]. Plasmid DNA was isolated using a GeneJET Plasmid Miniprep Kit (Thermo Fisher Scientific) or GeneMATRIX Plasmid Miniprep DNA Purification Kit (EURx, Gdańsk, Poland). Genomic DNA was isolated using a GeneMATRIX Bacterial & Yeast Genomic DNA Purification Kit (EURx). PCRs were performed with Phusion High-Fidelity DNA polymerase or DreamTaq DNA polymerase (Thermo Fisher Scientific). Oligonucleotide primers used for PCR were purchased from Genomed S.A. (Warsaw, Poland) and are listed in Table S2. For the purification of DNA fragments amplified by PCR or obtained by restriction digestion, a GeneMATRIX PCR/DNA Clean-Up Purification Kit (EURx) was used. DNA sequencing was performed by Eurofins Genomics (Ebersberg, Germany).

### 4.3. Generation of Null hfq and omrA Mutants in Y. enterocolitica Strains

Null *hfq* and *omrA* mutants of *Y. enterocolitica* Ye9N and AR4 (∆*ompR*) were produced by homologous recombination using constructs based on suicide plasmid pDS132 [103]. Appropriate overlap extension PCR products were generated to delete the *hfq* or *omrA* genes by allelic exchange at the native *Y. enterocolitica* loci, simultaneously introducing antibiotic resistance cassettes (Tp and Gm, respectively). Briefly, for each gene, three overlapping DNA fragments were PCR-amplified: fragment A (5′ fragment of *hfq* or *omrA* gene; Ye9 genomic DNA as the template), fragment B (3′ fragment of *hfq* or *omrA* gene; Ye9 genomic DNA as the template) and fragment C (Tp or Gm cassette; p34E-Tp or pBBR1MCS-5 as a template, lab collection). Mixtures of fragments A, B and C were then used as the template with pairs of flanking primers, dHfq(D)_F/dHfq(D)_R or dOmrA(D)_F/dOmrA(D)_R (Table S2), to generate the final PCR products. These DNA fragments were digested with XbaI (FastDigest, Thermo Fisher Scientific) and cloned into the corresponding site in pDS132. The obtained plasmid, pDShfq or pDSOmrA, was used to transform *E. coli* S17-1 λ*pir* with selection on Tp or Gm. Finally, the plasmids were conjugally transferred from the donor *E. coli* strains into *Y. enterocolitica* strains, and transconjugants were selected with Cm. Integration of the plasmid into the chromosome after a single cross-over was verified by PCR. Selection for the second recombination event was performed by plating onto selective medium (Tp or Gm) containing 10% (*w*/*v*) sucrose. Colonies from sucrose selection medium were tested for Cm sensitivity. Deletion mutants were verified using colony PCR with primers flanking the expected site of mutagenesis, and the amplified fragments were sequenced.

### 4.4. Construction of Plasmid-Borne gfp Translational Fusions with fepA and fecA

Translational fusions of *fepA* and *fecA* with a *gfp* reporter gene were constructed using the Golden Gate technique [104] in plasmid pFX-P, Sp^r^ [105]. DNA fragments carrying the promoter, 5′ untranslated region (5′ UTR) and several codons of the gene of interest (18 codons of *fecA* and 13 of *fepA*) were amplified by PCR. The primers used for this purpose contained Eco31I sites and additional sequences designed to generate compatible ends with Eco31I-digested pFX-P (Table S2). In a 20 μL Golden Gate cloning reaction, 50 ng of vector was mixed with 100 ng of PCR product, 5 units of Eco31I (BsaI isoschizomer, Thermo Fisher Scientific) and 4.5 units of T4 DNA ligase (Thermo Fisher Scientific) in ligase buffer. Each reaction was incubated for 5 min at 37 °C, 10 min at 22 °C (for 50 cycles), followed by 5 min at 50 °C and 10 min at 65 °C, and then it was used to transform *E. coli* DH5α. Clones were selected on LB agar supplemented with Sp, and their identities were confirmed by PCR and DNA sequencing. Plasmids pFX-*fecA* or pFX-*fepA* carrying the fusions were then introduced by electroporation into the desired *Y. enterocolitica* strains. The green fluorescence of cultures was assessed using a TECAN Infinite^®^ 200 PRO fluorometer (Tecan, Männedorf, Switzerland).

### 4.5. Construction of Strains Expressing FecA and FepA Carrying a 3×FLAG Epitope

The FLAG tag was fused to the C-terminus of recombinant FecA and FepA to facilitate protein detection. In-frame fusions of the *fecA* and *fepA* genes with the 3×FLAG epitope coding sequence were constructed by overlap extension PCR using primers listed in Table S2. Two overlapping DNA fragments (A and B) were PCR-amplified using *Y. enterocolitica* Ye9 genomic DNA as the template. Fragment A comprised 529 bp of the *fecA* gene without a STOP codon (or 605 bp of the *fepA* gene without a STOP codon) with an overhang of 20 bp representing the 1×FLAG coding sequence. Fragment B comprised the 3×FLAG coding sequence (69 bp) plus 175 bp downstream of the *fecA* STOP codon (or 582 bp downstream of the *fepA* STOP codon). Mixtures of amplicons A and B were then used as the template in PCR with pairs of flanking primers, 1FecAFLAGXba_F/4FLAGFecAXba_R or 1FepAFLAGXba_F/4FLAGFepAXba_R, to generate fragments of 761 bp and 1236 bp, respectively. These fragments were digested with XbaI and then cloned into the corresponding site of suicide vector pDS132 [103]. The resulting constructs pDSfecA-FLAG and pDSfepA-FLAG were verified by sequencing and then introduced into different *Y. enterocolitica* strains via conjugation from the donor *E. coli* S17-1 λ*pir*. Transconjugants carrying pDSfecA-FLAG or pDSfepA-FLAG integrated into the chromosome were selected with Cm. To force the second cross-over recombination, strains were plated on medium supplemented with 10% (w/v) sucrose. Sucrose-resistant colonies that had lost Cm resistance were screened for the 3×FLAG coding sequence using colony PCR with primer pair FlagSpr1/FlagSpr2. The presence of intact fusions carrying a 3×FLAG epitope peptide coding sequence was confirmed by sequencing the PCR products using appropriate primers (Table S2).

### 4.6. Western Blotting

*Y. enterocolitica* strains were grown under the desired conditions until OD_600_ ~ 1. Volumes of 1 mL of each culture were then centrifuged, and the cell pellets were resuspended in OD_600_ ×100 μL of Laemmli buffer [102]. Samples were then heated at 95 °C for 10 min to solubilize cellular proteins, which were separated by SDS-PAGE. All gels were prepared using a TGX Stain-Free FastCast Acrylamide Kit (12%, BioRad, Hercules, CA, USA). The loading of equivalent amounts of protein was verified by UV-induced fluorescence with a GE Healthcare AI600 Imager (GE Healthcare, Chicago, IL, USA). Then, proteins were electroblotted onto a nitrocellulose membrane (Amersham Protran 0.2 µm Western Blotting Membrane; GE Healthcare) using a wet electroblotting system (100 V for 1 h; Bio-Rad). After blocking with 3.5% non-fat dried milk diluted in TBST, the membranes were incubated with mouse anti-FLAG monoclonal antibody (1:5000, Merck, Darmstadt, Germany). Then, the blot was washed with TBST and incubated with sheep anti-mouse IgG conjugated with HRP secondary antibody (1:8000, Merck). After washing the blot, positive immunoreactions were visualized using Clarity Western ECL Blotting Substrate (Bio-Rad) for HRP-based chemiluminescent detection with a GE Healthcare AI600 Imager.

### 4.7. Construction of a P_omrA_::gfp Transcriptional Fusion in Plasmid pPROBE-TT’

To obtain a transcriptional P*_omrA_*::*gfp* fusion, a 133 bp DNA fragment representing the *omrA* promoter region (−134/−1) upstream of the translational start site and containing a predicted OmpR-binding motif was amplified by PCR with the primer pair OmrA1E/OmrA133K (Table S2) using *Y. enterocolitica* Ye9N genomic DNA as the template. The obtained fragment was then purified, digested with restriction endonucleases EcoRI and KpnI (FastDigest, Thermo Fisher Scientific), whose unique sites were included in the primers, and ligated with the mobilizable vector pPROBE-TT’ cleaved with the same enzymes (Table S1). In this vector, the *omrA* promoter fragment was inserted upstream of the *gfp* gene to form plasmid pPomrA. The obtained P*_omrA_*::*gfp* fusion was verified by PCR and DNA sequencing with the primers used to amplify the promoter fragment (Table S2). The plasmid pPomrA and the empty parent vector pPROBE-TT’ were introduced into *E. coli* CC118 λpir by transformation and then transferred into *Y. enterocolitica* strains by triparental conjugation using *E. coli* DH5α containing the plasmid pRK2013 carrying *tra* and *mob* genes [106]. The presence of these constructs in the *Y. enterocolitica* transformants was confirmed by plasmid isolation.

### 4.8. Measurement of GFP Fluorescence

To study the effect of regulator OmpR and stress factors on *omrA* gene expression, early log-phase bacterial cultures (OD_600_ ~ 0.2) expressing GFP were incubated for ~2 h at 26 °C or 37 °C and then exposed to different stress conditions for 60 min or incubated until they reached early stationary phase (OD_600_ ~ 1). Next, 200 μL of the cultures were transferred to wells of Corning^®^ 96 Well Black Polystyrene Microplates (black, clear bottom, Merck). Absorbance at 600 nm and GFP fluorescence (excitation 485 nm; emission 530 nm) were measured using a TECAN Infinite^®^ 200 PRO microplate reader. GFP fluorescence was expressed as the relative fluorescence intensity (RFU) divided by the OD_600_ after subtracting the values of a blank sample. A culture of strain Ye9N carrying vector pPROBE-TT’ [107] was used to determine background fluorescence. Individual cultures were assayed in triplicate, and the reported values are the means from three independent cultures.

### 4.9. Generation of a Plasmid Overexpressing ryhB1 and omrA from a Foreign Promoter

Plasmid pBR-plac carrying a modified inducible P_LlacO-1_ promoter [108], originally used to clone *omrA* and *omrB* from *E. coli*, was received from Prof. Susan Gottesman [24]. The presence of an AatII restriction site in pBR-plac between positions −6 and −1 relative to the transcription start site and a unique EcoRI restriction site made it possible to clone *ryhB1* and *omrA* of *Y. enterocolitica* Ye9N without including any possible regulatory sequences in the 5′ end. Oligonucleotides RyhB1-For and RyhB1-Rev or OmrA-For and OmrA-Rev were annealed, and the resulting duplexes were cloned into vector pBR-plac cleaved with AatII and EcoRI. *E*. *coli* DH5α was transformed with the ligation mixtures. The correctness of the insert in recombinant plasmid clones was confirmed by sequencing with two vector primers (pBR_F and pBR_R). The obtained constructs pBR-RyhB1 and pBR-OmrA were then introduced into *Y. enterocolitica* strains by electroporation. It should be noted that the expression of sRNAs from obtained plasmids in *Y. enterocolitica* did not require IPTG induction.

### 4.10. RNA Isolation and Northern Blot Analysis

The levels of transcripts were assessed by Northern blot analysis. Overnight cultures were diluted 100-fold in 10 mL of fresh medium and grown at 26 °C for about 6 h (until OD_600_ = 1). Total RNA was isolated using TRItidy™ (AppliChem, Darmstadt, Germany), as described previously [109]. The quality of the RNA was checked by gel electrophoresis and quantified using a Qubit 4 Fluorometer with a Qubit RNA IQ Assay Kit (Thermo Fisher Scientific). For each sample, 10 µg of total RNA was separated by denaturing polyacrylamide gel electrophoresis (6% acrylamide/bis 29:1, 7 M urea). The RNA was then electro-transferred to a Zeta-Probe^®^ Blotting Membrane (Bio-Rad, Hercules, CA, USA). Crosslinking was performed by UV irradiation (1200 J, UVP HL-2000 HybriLinker^TM^, Analytik Jena, Jena, Germany). The membrane was then incubated with DNA oligonucleotide probes labeled with ^32^P (Table S2). After washing to remove unbound probes, membranes were exposed to phosphor storage screens and analyzed using a Typhoon FLA 9000 biomolecular imager (GE Healthcare). Quantification was performed using ImageQuantTL (version 8.2, GE Healthcare). The presented blots are representative of at least two independent experiments.

### 4.11. RT-qPCR

Total RNA was isolated from cultures of *Y. enterocolitica* strains grown in LBD medium at 26 °C (each culture in triplicate). After the cultures had reached OD_600_ ~ 1, the cells were harvested by centrifugation, and RNA was isolated from the cell pellets using a Nucleo Spin RNA purification kit (Macherey-Nagel, Düren, Germany). RNA samples were treated with a TURBO DNA-free^TM^ kit (Invitrogen Waltham, MA, USA) to ensure the complete removal of contaminating DNA. The purity and quality of the RNA were assessed using a Qubit 4 fluorometer with a Qubit RNA IQ Assay Kit (Thermo Fisher Scientific). First-strand cDNA synthesis was performed using a Maxima H Minus First-Strand cDNA Synthesis Kit (Thermo Fisher Scientific). Real-time PCR was performed using 5x HOT FIREPol EvaGreen qPCR Mix Plus (Solis Biodyne, Tartu, Estonia) with a LightCycler 96 System (Roche, Basel, Switzerland). Oligonucleotide primers used for qPCR were purchased from Genomed S.A. (Warsaw, Poland) and are listed in Table S2. The levels of the amplified PCR products were normalized to that of a fragment amplified from the 16S rRNA reference gene. Fold changes were calculated using the Pfaffl method [110].

### 4.12. Electrophoretic Mobility Shift Assay (EMSA)

The binding of phosphorylated OmpR-His_6_ to the regulatory region of the *omrA* gene containing a putative OmpR-binding site was examined by the EMSA technique. Overproduction and purification of recombinant OmpR-His6 for in vitro DNA binding studies were performed as described previously [74]. Briefly, *Y. enterocolitica* OmpR protein with a His_6_ motif at its N-terminus was expressed from plasmid pETOmpR in *E. coli* BL21(DE3) and purified using Ni-NTA resin (Qiagen, Hilden, Germany). The concentration of the purified OmpR-His6 was determined using the RC DC protein assay (Bio-Rad). A 250 bp DNA fragment comprising the regulatory region upstream of *omrA* was amplified by PCR with primer pair EomrAYe_F/EomrAYe_R (Table S2), with Ye9N genomic DNA as the template. A fragment of 16S rDNA (304 bp, amplified using primers E16S304Ye_F/E16S304Ye_R, Table S2) was prepared for use as a non-specific binding control. The amplicons were purified using a GeneMatrix PCR/DNA Clean-Up Kit (EURx), and the DNA concentrations were determined with a NanoDrop 2000 spectrophotometer (Thermo Fisher Scientific). The process of in vitro OmpR-His_6_ phosphorylation by incubation with acetyl phosphate and binding of His-tagged OmpR-P was conducted as described previously [22,111]. The binding reactions contained the 250 bp *omrA* promoter fragment (0.3 pmol) and 304 bp 16S rDNA control fragment (0.3 pmol) mixed with increasing amounts of OmpR-P (12.4–74.6 μM) in OmpR-binding buffer (50 mM Tris-HCl pH 8.0, 100 mM KCl, 1 mM EDTA, 1 mM DTT, 20 mM MgCl_2_, 12% glycerol, 100 µg/mL BSA, 0.1% Triton X-100). OmpR-P binding to the DNA target was performed at 22 °C for 15 min. The OmpR-P/DNA complexes were separated by electrophoresis in 6% polyacrylamide gels under non-denaturing conditions (19:1 acrylamide/bisacrylamide, 0.2×TBE containing 2% glycerol) in 0.2×TBE running buffer for 3 h at 110 V. After staining the gels in 1×SYBRgreen solution (Invitrogen), DNA-containing bands were visualized using a GE Healthcare AI600 Imager.

### 4.13. Bioinformatic and Statistical Analyses

In silico analyses were performed on the *Y. enterocolitica* Ye9N (bioserotype 2/O:9) *omrA* locus obtained from a shotgun genome sequence (NCBI/GenBank: NZ_JAALCX010000053.1). Other bioinformatic analyses examined complete genome sequences available through the NCBI (https://www.ncbi.nlm.nih.gov/genbank/, accessed on 23 January 2023): *Escherichia coli* str. K-12 substr. MG1655 (NCBI tax. ID: 511145); *Shigella flexneri* serotype 2a str. 301 (NCBI tax. ID: 198214); *Salmonella enterica* subsp*. enterica* serovar Typhimurium str. 14028S (NCBI tax. ID: 588858); *Klebsiella pneumoniae* subsp*. pneumoniae* HS11286 (NCBI tax. ID: 1125630); *Y. enterocolitica* subsp. *palearctica* 8081 (NCBI tax. ID: 393305); *Y. intermedia* (NCBI tax. ID: 631); *Y. ruckeri* ATCC 29473 (NCBI tax. ID: 527005); *Y. pseudotuberculosis* IP 32953 (NCBI tax. ID: 273123); *Y. pestis* str. A1122 (NCBI tax. ID: 1035377); *Serratia marcescens* strain KS10 (NCBI tax. ID: 615); *Erwinia carotovora* subsp. *atroseptica* SCRI1043 (NCBI tax. ID: 218491); *Dickeya dadantii* 3937 (NCBI tax. ID: 198628). The program T-Coffee [79] was used for sequence alignments. For sequence alignment editing, visualization, analysis and phylogenic tree generation, the Jalview program was used [80]. A heatmap was created using MATLAB R2021b version 9.11 (https://se.mathworks.com/, accessed on 23 January 2023). Complementary nucleotide sequences in sRNAs RyhB or OmrA and mRNAs were identified using IntaRNA software version 5.0.7 (http://rna.informatik.uni-freiburg.de/IntaRNA, accessed on 23 January 2023). Secondary structures of mRNAs were predicted with UNAFold software version 3.5 (http://www.unafold.org/mfold/applications/rna-folding-form.php, accessed on 23 January 2023). Homology searches were performed with BLAST software version 2.9.0 (https://blast.ncbi.nlm.nih.gov/Blast.cgi, accessed on 23 January 2023) and Clustal Omega version 1.2.4 *(*https://www.ebi.ac.uk/Tools/msa/clustalo/, accessed on 23 January 2023). The diagram of the phylogenetic tree was created with Jalview version 2.8.2. Statistical analyses were performed using Prism 7 software (v.7.02, GraphPad, San Diego, CA, USA). One-way ANOVA or Student’s unpaired t-test was used to determine statistically significant differences.

## 5. Conclusions

*Y. enterocolitica* faces several different challenges during infection and colonization of the human body. Acquisition of iron is necessary for growth within the host, while on the other hand, its intracellular accumulation can be toxic for bacteria. Thus, tight regulation of iron acquisition is essential to fulfill the iron requirements of a pathogen while preventing harmful over-accumulation. The results of this study reveal that Hfq-mediated interaction of certain sRNAs with their target mRNAs (at least *fur*, *fecA* and *fepA*) might regulate diverse iron acquisition mechanisms, with the exact strategy tailored to conditions in specific ecological niches, including the availability of iron and siderophores in the host body. The tight regulation of FecA and FepA by a coherent feed-forward OmpR/sRNA OmrA regulatory loop may be a vital strategy for growth/survival in niches that vary in several physico-chemical parameters. High osmolarity and low pH, conditions characteristic of the gut environment and known to activate/phosphorylate OmpR, may induce the expression of OmrA, which acts to reduce the synthesis of the iron transporters FepA and FecA to prevent the acquisition of too much iron. The solubility of iron is known to increase in acidic conditions; therefore, the uptake of ferric siderophores by FepA and FecA receptors might be less necessary under these circumstances. It is noteworthy that post-transcriptional mechanisms that reduce the abundance of FecA and FepA may help *Y. enterocolitica* to evade the host’s immune surveillance by decreasing the levels of immunogenic epitopes in the outer membrane. Given the complexity of the OmpR and Fur regulatory circuits, post-transcriptional regulatory networks (Hfq/sRNAs) may be significant and contribute to the fitness of *Y. enterocolitica.*

## Data Availability

The data that support the findings of this study are available from the corresponding author upon reasonable request.

## Supplementary Materials

The following supporting information can be downloaded at: https://www.mdpi.com/article/10.3390/ijms241311157/s1.
