# Supplementary material for: Interplay between the RNA Chaperone Hfq, Small RNAs and Transcriptional Regulator OmpR Modulates Iron Homeostasis in the Enteropathogen Yersinia enterocolitica
Source: Int J Mol Sci. 2023 Jul 6;24(13):11157. doi: 10.3390/ijms241311157 (PMC10342277; doi:10.3390/ijms241311157)
Supplement: Supplementary file 1 [file ijms-24-11157-s001.zip › ijms-2486748-supplementary.pdf]

Table S1. Strains and plasmids used in this study

Strains and plasmids	Description	Reference or source
***Y. enterocolitica* 2/O:9 strains**		
Ye9	Clinical isolate of serotype O:9, carrying virulence plasmid pYV	Clinical isolate, laboratory collection
Ye9N	Ye9 derivative, spontaneous Nal^R mutant	[1]
AR4	Ye9N derivative Δ*ompR*::Km defective in OmpR production, Nal^R, Km^R	[2]
Ye9*hfq*	Ye9N derivative Δ*hfq*::Gm defective in Hfq production, Nal^R, Gm^R	This study
Ye9*omrA*	Ye9N derivative Δ*omrA*::Gm defective in OmrA synthesis, Nal^R, Gm^R	This study
Ye9Fflag	Ye9N, *fur*::3×*flag*, Nal^R	[3]
Ye9FecAflag	Ye9N, *fecA*::3×*flag*, Nal^R	This study
Ye9FepAflag	Ye9N, *fepA*::3×*flag*, Nal^R	This study
***E. coli* strains**		
DH5α	F′, *endA1*, *hsdR17*(r^-_k m^+_k), *supE44*, *thi-1*, *recA1*, Δ(*lacIZYA-argF*) *U169 deoR* [ϕ80dlacΔ(*lacZ*)M15]	[4]
TOP 10F′	F'{*lac*I^qTn10(Tet^R)} *mcr*A Δ(*mrr-hsd*RMS-*mcr*BC) φ80*lac*ZΔM15 Δ*lac*X74 *rec*A1 *ara*D139 Δ(*ara-leu*)7697 *gal*U *gal*K *rps*L *end*A1 *nup*G	Thermo Fisher Scientific
S17-1 *λpir*	Tp^R, Str^R, *pro*, *thi*, *recA*, *hsdR514*, (r^- m^+), *λpir*, RP4: 2- Tc::Mu-Km^R::Tn7	[5]
CC118 *λpir*	Δ(*ara-leu*) *araD* Δ*lacX174 galE galK phoA thiE1 rpsE rpoB*(Rif^R) *argE*(Am) *recA1*, *λpir* lysogen	[6]
BL21 (DE3)	F^- , *ompT hsdSB* ($r_{B-}m_{B-}$) *gal*, *dcm* (DE3)	Life Technologies
**Plasmids**		
pFX-P	Golden Gate-compatible pDSK602 derivative without promoter for generation of translational mRNA::*gfp* fusions, Sp^R	[7]
pFX-*fur*	pFX-P derivative carrying untranslated region of *fur* (5'UTR) with the first 8 codons of ORF *fur*, fused in frame with *gfp*, Sp^R	O. Rossier laboratory
pFX-*fecA*	pFX-P derivative carrying untranslated region of *fecA* (5'UTR) with the first 18 codons of ORF *fecA*, fused in frame with *gfp*, Sp^R	This study
pFX-*fepA*	pFX-P derivative carrying untranslated region of *fepA* (5'UTR) with the first 13 codons of ORF *fepA* fused in frame with *gfp*, Sp^R	This study
pFX-*ompR*	pFX-P derivative carrying untranslated region of *ompR* (5'UTR) with the first 4 codons of ORF *ompR* fused in frame with *gfp*, Sp^R	[8]
pBR-plac (pBR1)	pBR322 with SspI/AatII artificial $P_{LlacO-1}$ promoter modified by the introduction of an AatII restriction site between position -6 and -1 relative to the transcription start site, Tc^R	[9]

pBR-RyhB1	pBR1 with AatII/EcoRI *ryhB1* sequence, Tc^R	This study
pBR-OmrA	pBR1 with AatII/EcoRI *omrA* sequence, Tc^R	This study
pHR4	pHSG575 with 740 bp fragment containing entire coding sequence of *ompR* (ORF with rbs), Cm^R	[2]
pRK2013	Helper plasmid, Km^R	[10]
pDS132	*ori* R6K (narrow host range, replication only in *E. coli λpir*), *oriT* RK2, *sacB*, Cm^R	[11]
pDSomrA	pDS132 derivative carrying 2135 bp cassette for *omrA* mutagenesis constructed by overlap extension PCR cloned between XbaI sites of the vector, Gm^R	This study
pDShfq	pDS132 derivative carrying 1756 bp cassette for *hfq* mutagenesis constructed by overlap extension PCR cloned between XbaI sites of the vector, Tp^R	This study
pPROBE TT′	Broad-host-range cloning vector pBBR1MCS-3 with promoterless *gfp*, Tc^R	[12]
pPomrA	derivative of pPROBE TT′ with subcloned 149 bp EcoRI/KpnI fragment of *omrA* promoter region, Tc^R	This study
pDSfur-FLAG	pDS132 carrying 132 bp upstream of *fur* start codon and 444 bp of *fur* ORF (without stop codon) fused in frame with 69 bp of 3×FLAG epitope and 663 bp downstream of *fur* stop codon	This study
pDSfecA-FLAG	pDS132 carrying 529 bp fragment of *fecA* gene without a STOP codon fused in frame with 69 bp of 3×FLAG epitope and 175 bp downstream of *fecA* stop codon	This study
pDSfepA-FLAG	pDS132 carrying 605 bp fragment of *fepA* gene without a STOP codon fused in frame with 69 bp of 3×FLAG epitope and 582 bp downstream of *fecA* stop codon	This study
pBAD24Cm	pBAD24 with CAT (Cm^r) replacing *bla*, *ori* pBR322, MCS-3, *araC*	[13]
pBAD-Hfq	pBAD24 with 306-bp fragment of *hfq* gene	O. Rossier laboratory
Cm^R chloramphenicol resistance, Gm^R gentamicin resistance, Km^R kanamycin resistance, Nal^R nalidixic acid resistance, Sm^R streptomycin resistance, Tc^R tetracycline resistance, Tp^R trimethoprim resistance, ::Km, insertion of kanamycin resistance cassette; ::Gm, insertion of gentamicin resistance cassette		

Table S2. Oligonucleotide primers used in this study

Purpose and Target	Name of primer	Primer sequence (5′→ 3′)	Restriction sites	Reference
Construction of Δ*omrA* mutant	dOmrA(A)_F	GCTCTAGACAGCGGCATCTGTCTTATTC	XbaI	This study
	dOmrA(A)_R	CATCCGTTTCCACGCACTACTCTATCCG		
	dOmrA(B)_F	CGGATAGAGTAGTGCGTGGAAACGGATG		
	dOmrA(B)_R	GCGGAGAGAACAAACGATCTCGGCTTGA		
	dOmrA(C)_F	TCAAGCCGAGATCGTTTGTTCTCTCCGC		
	dOmrA(C)_R	CGTCTAGAATGGCGTGTAAGCGCTCACC	XbaI	
	dOmrA(D)_F	TGGGTGCCGTTGACGGATTG		
	dOmrA(D)_R	GGCCAGGGTCAGACTTTCTC		
Construction of Δ*hfq* mutant	dHfq(A)_F	CGTCTAGAGGTAAAACTCTAACAGAACTGAC	XbaI	This study
	dHfq(A)_R	TGTCAACTGGGTTCGTGAATTCTCTATATTTTCCTTATTTGCTTGTTGT		
	dHfq(B)_F	CAAGCAAATAAGGAAAATATAGAGAATTCACGAACCCAGTTGACA		
	dHfq(B)_R	TGACCAGCAATGCGCTGAATTCTTAGGCCACACGTTCAA		
	dHfq(C)_F	TTGAACGTGTGGCCTAAGAATTCAGCGCATTGCTGGTCA		
	dHfq(C)_R	CGTCTAGATACCAAACGAGTCGCAATAT	XbaI	
	dHfq(D)_F	TAATGATCCCCAACGGCTCT		
	dHfq(D)_R	GTTTCACCTGGCCCTCTTAG		
Construction of *fecA′*::*′gfp* translational fusion	FecAYe9_F	TTTGGTCTCTATTCCCCCCTTATTCCAAATGGTTTTTATTT	BsaI	This study
	FecAYe9_R	TTTGGTCTCTTAGCTAATGCCAGCGCGACGGA	BsaI	
Construction of *fepA′*::*′gfp* translational fusion	FepAYe9_fw	TTTGGTCTCTATTCGGCATAATGACTCCTTCACTGG	BsaI	This study
	FepAYe9_rev	TTTGGTCTCTTAGCTAAGGTCGTCAGAGAGCGCC	BsaI	
Confirmation of *gfp* sequence	gfp_F	AGTGGAGAGGGTGAAGGTGA		This study
	gfp_R	AAAGGGCAGATTGTGTGGAC		
Confirmation of the correct sequence of translational fusions	OR177pFX_fw	CCATGCTCAGAAAAGGCTTAACA		[7]
	OR178pFX_rev	CCGTATGTAGCATCACCTTCA		
Construction of P_{omrA}::*gfp* transcriptional fusion	omrAE_F	TGGAATTCATCGGATGTACCGCAATGA	EcoRI	This study
	omrAK_R	TGGGTACCGGGATCACTACTCTATCCGCTTA	KpnI	
Confirmation of the correct sequence of P_{omrA}::*gfp* transcriptional fusion	pBR_F	ACCGCTGTTGAGATCCAGTT		This study
	pBR_omrA_R	CGTAGGTCGGTGCAAATAAA		
Construction of pBR-RyhB1 overexpression plasmid	RyhB1-For	GACGTCGCTTTCAGATGAGACCATCAAAGTTTAGGTGTTCACATTACGAA GGCACGACATTGCTCACATTGCTTCCAGTGTTTACTTAGCCAGCCGGGTG CTGGCTTTTGAATTC	AatII, EcoRI	This study

	RyhB1-Rev	AATTCAAAAGCCAGCACCCGGCTGGCTAAGTAAACACTGGAAGCAATGTGAGCAATGTCGTGCCTTCGTAATGTGAACACCTAAACTTTGATGGTCTCATCTGAAAGCGACGT	EcoRI, AatII	
Construction of pBR-OmrA overexpression plasmid	OmrA-For	GACGTCCCCAGAGGTATTAATTGGTGAGTAATCAACATACGCTGTGTGTTAAAGCCAGTTTTTTATTTGCACCGACCTACGCAGATGCGTAGGTTTTTTTTGGAATTC	AatII, EcoRI	This study
	OmrA-Rev	AATTCCAAAAAAAACCTACGCATCTGCGTAGGTCGGTGCAAATAAAAAACTGGCTTTAACACACAGCGTATGTTGATTACTCACCAATTAATACCTCTGGGGACGT	EcoRI, AatII	
Confirmation of the correct sequence of pBR- overexpression vectors	pBR1-For	TAGTGTATGCGGCGACCGAG		This study
	pBR1-Rev	ACGGTGCCTGACTGCGTTAG		
Construction of strains carrying FecA-3×FLAG	1FecFLAGXba-F	GCTCTAGAACAAATCTGGGAGCGACAAC	XbaI	This study
	2FecFLAG-R	CCGTCATGGTCTTTGTAGTCGAAGGCAACTGACCCCTGC		
	3FLAGFec-F	GACTACAAAGACCATGACGGTGATTATAAAGATCATGATATCGATTACA-AGGATGACGATGACAAGTAGATTAGCAGGTTAAGATAAGCC		
	4FLAGFecXba-R	GCTCTAGATCACTGGCTCATCTGTTGGT	XbaI	
	FlagSpr1	TCATCGTCATCCTTGTAATCG		
	FlagSpr2	CTACAAAGACCATGACGGTGA		
	5FecFLAG-F	TGGGTCTGGAACCTGGATAG		
	6FecFLAG-R	AATACCTAAGCCCGGCAAAT		
Construction of strains carrying FepA-3×FLAG	1FepFLAGXba-F	GCTCTAGACCGTAATAAGATTGAGCCAGGT	XbaI	This study
	2FepFLAG-R	CCGTCATGGTCTTTGTAGTCAAACTGGGTATTCAAGCTAACAAAA		
	3FLAGFep-F	GACTACAAAGACCATGACGGTGATTATAAAGATCATGATATCGATTACA-AGGATGACGATGACAAGTAGCCGTTAGGAAAATCACCTAAAAATAG		
	4FLAGFepXba-R	GCTCTAGACACGACTCTCTCCTCGCTTT	XbaI	
	FlagSpr1	TCATCGTCATCCTTGTAATCG		
	FlagSpr2	CTACAAAGACCATGACGGTGA		
	5FepFLAG-F	CCGGTGCAGCAACCTATAAT		
	6FepFLAG-R	AACCTCCCCGCATAATAACC		
EMSA, 250 bp fragment of *omrA*	EomrAYe_F	GAGTTATCGAGGTTTGTCAGCA		This study
	EomrAYe_R	TGGCTTTAACACACAGCGTA		
EMSA, 304 bp fragment of 16S rDNA used as a negative control	E16S304Ye_F	ATTCCGATTAACGCTTGCAC		[14]
	E16S304Ye_R	GTGGGGTAATGGCTCACCTA		
RT-qPCR analysis of *Y. enterocolitica fur*	RT*fur*Ye9_F	CGGTATTGTTACCCGCCATAA		[3]

expression	RT*fur*Ye9_R	TCACTTTGCCGCAATCCA		
RT-qPCR analysis of *Y. enterocolitica fecA* expression	RT*fecA*Ye9_F	CTTGACGGCTGAAAAAGCACA		[3]
	RT*fecA*Ye9_R	TGAATGCCAACTCCACACCT		
RT-qPCR analysis of *Y. enterocolitica fepA* expression	RT*fepA*Ye9_F	ATGCGGTGCGTTATGGTTG		[3]
	RT*fepA*Ye9_R	TGATATTCACCACGCCACCT		
RT-qPCR analysis of *Y. enterocolitica* gene expression – internal control 16s rRNA	RT16rRNAYe9-F	CATCATGGCCCTTACGAGTAG		[3]
	RT16rRNAYe9-R	CCGGACTACGACAGACTTTATG		
RyhB-1 probe for Northern blot	RyhB-1 NB	TCGTAATGTGAACACCTAAACTTTGATGGT		This study
OmrA probe for Northern blot	OmrA NB	GTGCAAATAAAAAACTGGCTTTAACACACA		This study

Table S3. sRNA OmrA- and OmrB-encoding genes in selected Gammaproteobacteria

Species[a]	sRNA gene	Strand[b]	Start[c]	End[c]	Adjacent genes[d]	Orientation[e]
E.col.	***omrA***	-	2976189	2976102	*aas/omrB*	<<<
S.fle.		-	2936548	2936461	*aas/omrB*	<<<
S.ent.		-	3190017	3189931	*aas/omrB*	<<<
K.pne.		-	4346498	4346411	*aas/omrB*	<<<
***Y.ent.* Ye9N**[f]		NA	NA	NA	*ISYen1 family transposase/aas*	NA
***Y.ent.* 8081**		-	3638400	3638306	*aas/ISYen1 family transposase*	<<>
Y.int.		+	828423	828517	*bisC/aas*	>>>
Y.ruc.		-	2931527	2931435	*aas/hp*	<<>
Y.pse.		+	4047527	4047621	*bisC/aas*	>>>
Y.pes.		+	233753	233847	*bisC/aas*	>>>
S.mar.		+	578519	578611	*bisC/aas*	>>>
E.car.		-	4090604	4090514	*aas/gene encoding methyl-accepting chemotaxis protein*	<<>
D.dad.		-	3953607	3953517	*bglG/lpxO*	<<<
E.col.	***omrB***	-	2976385	2976304	*omrA/galR*	<<>
S.fle.		-	2936745	2936664	*omrA/galR*	<<>
K.pne.		-	4346696	4346615	*omrA/galR*	<<>
S.ent.		-	3190217	3190133	*omrA/galR*	<<>

[a]Selected species: *Escherichia coli* str. K-12 substr. MG1655 (*E.col.*; NCBI:taxid511145); *Shigella flexneri* serotype 2a str. 301 (*S.fle.*; NCBI:taxid198214); *Salmonella enterica* subsp. *enterica* serovar Typhimurium str. 14028S (*S.ent.*; NCBI:taxid588858); *Klebsiella pneumoniae* subsp. *pneumoniae* HS11286 (*K.pne.*; NCBI:taxid1125630); *Yersinia enterocolitica* subsp. *palearctica* Ye9N bioserotype 2/O:9 (*Y.ent* Ye9N; a shotgun genome sequence: Accession number JAALCX010000053.1); *Yersinia enterocolitica* subsp. *enterocolitica* 8081 bioserotype 1B/O:8 (*Y.ent.* 8081; NCBI:taxid:393305); *Yersinia intermedia* (*Y.int.*; NCBI:taxid631); *Yersinia ruckeri* ATCC 29473 (*Y.ruc.*; NCBI:taxid527005); *Yersinia pseudotuberculosis* IP 32953 (*Y.pse.*; NCBI:taxid273123); *Yersinia pestis* str. A1122 (*Y.pes.*; NCBI:taxid1035377); *Serratia marcescens* strain KS10 (*S.mar.*; NCBI:taxid615); *Erwinia carotovora* subsp. *atroseptica* SCRI1043 (*E.car.*; NCBI:taxid218491); *Dickeya dadantii* 3937 (*D.dad.*; NCBI:taxid198628).

[b]The strand (+: forward strand; -: reverse strand) of the chromosome on which the sRNA is encoded.

[c]The specific location of the gene within the chromosome.

[d]The flanking regions upstream/downstream of the sRNA gene

[e]The orientation of the sRNA gene and of the adjacent left and right flanking genes
[f] NA - not assigned, *ISYen1 family transposase/omrA/aas* are localized in contig 53 (Accession number NZ_JAALCX010000053.1) of the *Yersinia enterocolitica* subsp. *palearctica* Ye9N bioserotype 2/O:9 genome

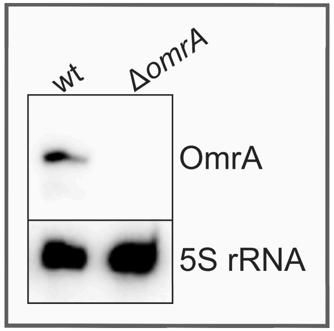

Figure S1. The abundance of *omrA* transcripts assessed by Northern blotting. The level of the *omrA* mRNA in the wild-type strain Ye9N and Δ*omrA* mutant (Ye9*omrA*) grown in LB medium were analyzed. As a loading control, the level of 5S rRNA was examined. The RNA molecules were detected by hybridization with ^{32}P-labeled DNA oligonucleotide probes.

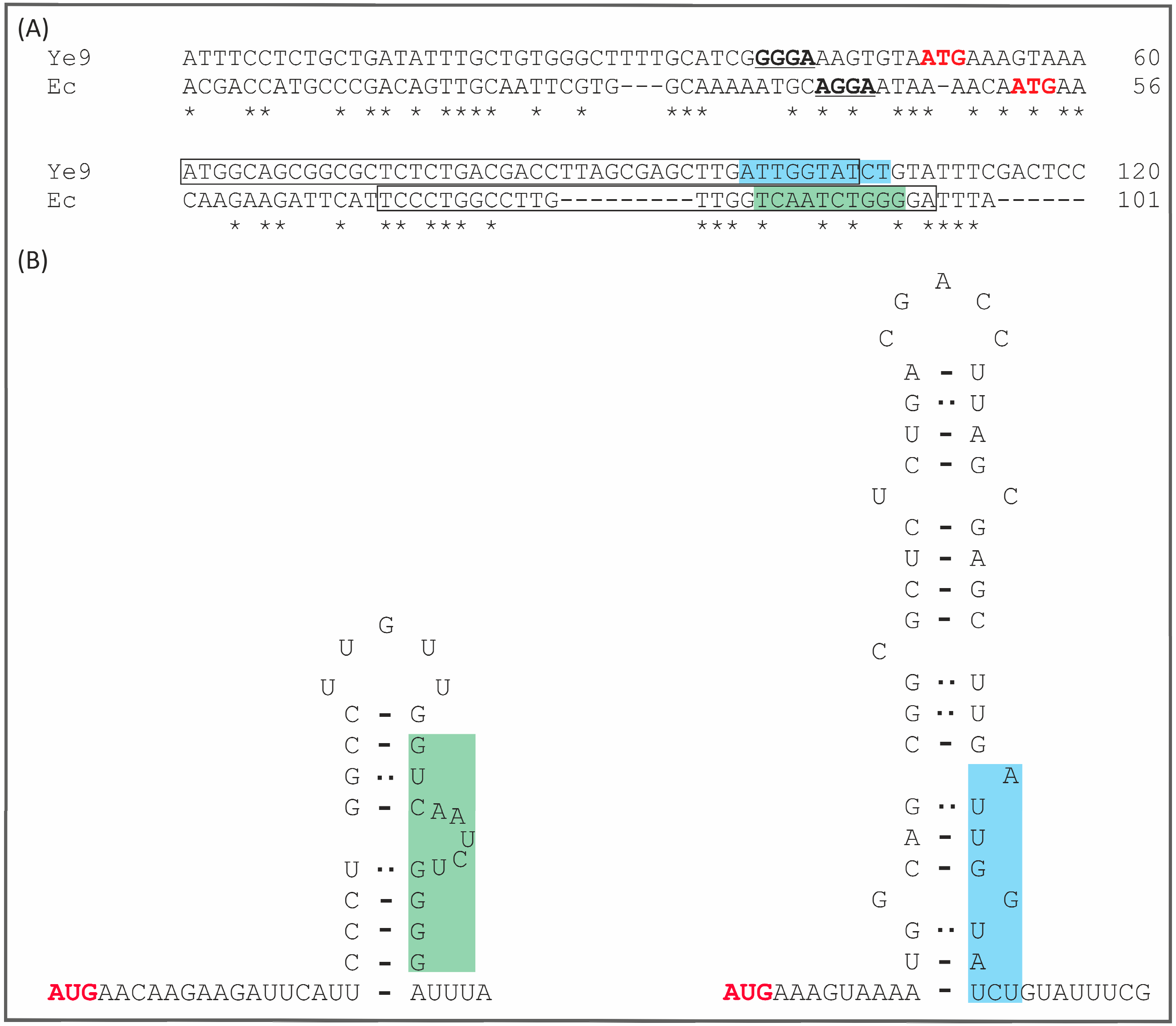

Figure S2. Predicted stem-loop like structures within *fepA* mRNAs and potential base-pairing with OmrA. (**A**) Sequence alignment of the 5′ UTRs and start of the coding regions of *fepA* from *Y. enterocolitica* and *E. coli*. The stem-loop like structures are boxed. Sites of predicted base pairing between the *fepA* mRNA and OmrA are highlighted in green for *E. coli* and blue for *Y. enterocolitica*. Conserved nucleotides are indicated by asterisks. (**B**) Predicted stem-loop like structures of *fepA* mRNAs of *E. coli* (left) and *Y. enterocolitica* (right), and the regions potentially targeted by OmrA. The alignment was performed using Clustal Omega. Secondary structures and possible base-paring were predicted using UNAFold (*http://www.unafold.org/mfold/applications/rna-folding-form.php*) and IntaRNA (*http://rna.informatik.uni-freiburg.de/IntaRNA*), respectively.

REFERENCES

1. Brzostek, K.; Brzostkowska, M.; Bukowska, I.; Karwicka, E.; Raczkowska, A. OmpR negatively regulates expression of invasin in *Yersinia enterocolitica*. *Microbiology* **2007**, *153*, 2416–2425, doi:10.1099/mic.0.2006/003202-0.
2. Brzostek, K.; Raczkowska, A.; Zasada, A. The osmotic regulator OmpR is involved in the response of *Yersinia enterocolitica* O:9 to environmental stresses and survival within macrophages. *FEMS Microbiol. Lett.* **2003**, *228*, 265–271, doi:10.1016/S0378-1097(03)00779-1.
3. Jaworska, K.; Ludwiczak, M.; Murawska, E.; Raczkowska, A.; Brzostek, K. The regulator OmpR in *Yersinia enterocolitica* participates in iron homeostasis by modulating Fur level and affecting the expression of genes involved in iron uptake. *Int. J. Mol. Sci.* **2021**, *22*, 1475. doi: 10.3390/ijms22031475.
4. Sambrook, J.; Russel, D. *Molecular Cloning: A Laboratory Manual*; 3rd ed.; Cold Spring Harbor Laboratory Press.: Cold Spring Harbor, NY, 2001
5. Simon, R.; Priefer, U.; Pühler, A. A Broad Host Range Mobilization System for *In Vivo* Genetic Engineering: Transposon Mutagenesis in Gram Negative Bacteria. *Bio/Technology* **1983**, *1*, 784–791, doi:10.1038/nbt1183-784.
6. Herrero, M.; de Lorenzo, V.; Timmis, K.N. Transposon vectors containing non-antibiotic resistance selection markers for cloning and stable chromosomal insertion of foreign genes in Gram-negative bacteria. *J. Bacteriol.* **1990**, *172*, 6557–6567.
7. Schmidtke, C.; Abendroth, U.; Brock, J.; Serrania, J.; Becker, A.; Bonas, U. Small RNA sX13: a multifaceted regulator of virulence in the plant pathogen *Xanthomonas*. *PLoS Pathog.* **2013**, 9:e1003626. 10.1371/journal.ppat.1003626.
8. Kakoschke, T.K.; Kakoschke, S.C.; Zeuzem, C.; Bouabe, H.; Adler, K.; Heesemann, J.; Rossier, O. The RNA chaperone Hfq is essential for virulence and modulates the expression of four adhesins in *Yersinia enterocolitica*. *Sci. Rep.* **2016**, *8*, 6:29275. doi: 10.1038/srep29275.
9. Guillier, M.; Gottesman, S. Remodelling of the *Escherichia coli* outer membrane by two small regulatory RNAs. *Mol. Microbiol.* **2006**, *59(1)*, 231-47. doi: 10.1111/j.1365-2958.2005.04929.x.
10. Figurski, D.H.; Helinski, D.R. Replication of an origin-containing derivative of plasmid RK2 dependent on a plasmid function provided in trans. *Proc. Natl. Acad. Sci. U. S. A.* **1979**, *76*, 1648–1652, doi:10.1073/pnas.76.4.1648.
11. Philippe, N.; Alcaraz, J.-P.; Coursange, E.; Geiselmann, J.; Schneider, D. Improvement of pCVD442, a suicide plasmid for gene allele exchange in bacteria. *Plasmid* **2004**, *51*, 246–255, doi:10.1016/j.plasmid.2004.02.003.
12. Miller, W.G.; Leveau, H.J.; Lindow, S.E. Improved *gfp* and *inaZ* Broad-Host-Range promoter-probe vectors. *MPMI* **2000**, 13, 1243–1250.
13. Guzman, L.M.; Belin, D.; Carson, M.J.; Beckwith, J. Tight regulation, modulation, and high-level expression by vectors containing the arabinose PBAD promoter. *J Bacteriol.* **1995**, *177*, 4121-4130, doi: 10.1128/jb.177.14.4121-4130.1995.
14. Nieckarz, M.; Raczkowska, A.; Debski, J.; Kistowski, M.; Dadlez, M.; Heesemann, J.; Rossier, O.; Brzostek, K. Impact of OmpR on the membrane proteome of *Yersinia enterocolitica* in different environments: repression of major adhesin YadA and heme receptor HemR. *Environ. Microbiol.* **2016**, *18*, 997–1021, doi:10.1111/1462-2920.13165.
